# Metallic 1T Na_*x*_MoS_2_ as Sulfur Host for Room Temperature Na–S Batteries

**DOI:** 10.1021/acsnano.6c04780

**Published:** 2026-05-20

**Authors:** Esther Lilian Gray, Jung-In Lee, Alice Elizabeth Beardmore, Leyi Loh, Ziwei Jeffrey Yang, Yan Wang, Manish Chhowalla

**Affiliations:** † Department of Materials Science and Metallurgy, 2152 University of Cambridge, Cambridge CB3 0FS, United Kingdom; ‡ The Faraday Institution, Quad One, Becquerel Avenue, Harwell Campus, Didcot OX11 0RA, United Kingdom; § Yusuf Hamied Department of Chemistry, University of Cambridge, Cambridge CB2 1EW, United Kingdom

**Keywords:** MoS_2_, Sodium intercalation, 1T phase, Raman mapping, XPS mapping, ^23^Na ssNMR, Na−S batteries

## Abstract

Sodium intercalation in layered materials is important for beyond lithium ion-based energy storage technologies. We show that the intercalation of sodium ions using a solid-state reaction between layered molybdenum disulfide (MoS_2_) and sodium borohydride (NaBH_4_) at 300 °C in an argon atmosphere leads to a transformation from the semiconducting to metallic phase and the formation of sodium-intercalated MoS_2_ (Na_*x*_MoS_2_). However, the removal of borohydride by washing with water also dissolves the intercalated Na ions, resulting in nonsodiated 1T phase MoS_2_. Therefore, 1T phase Na_*x*_MoS_2_ has been difficult to isolate and thus has remained largely unexplored. Here, we show that sequential washing with dimethylformamide (DMF) leads to the effective removal of borohydride while retaining intercalated sodium ions. This process yields stabilized Na_*x*_MoS_2_ (*x* ≈ 0.6 as determined by electrochemical deposition) with a 1T phase concentration of ∼60% measured by X-ray photoelectron spectroscopy (XPS). High resolution transmission electron microscopy (HR-TEM) results show that the 1T phase is uniformly distributed within the flake. ^23^Na magic-angle-spinning solid-state nuclear magnetic resonance (^23^Na ssNMR) confirms the interlayer intercalation of sodium ions. Finally, we employ 1T Na_*x*_MoS_2_ as a sulfur host in Na–S batteries. Cathodes based on 1T Na_*x*_MoS_2_ deliver higher specific capacity and improved cycling stability compared to conventional carbon/sulfur cathodes. These results suggest that 1T Na_*x*_MoS_2_ is promising for room temperature Na–S batteries.

## Graphical abstract

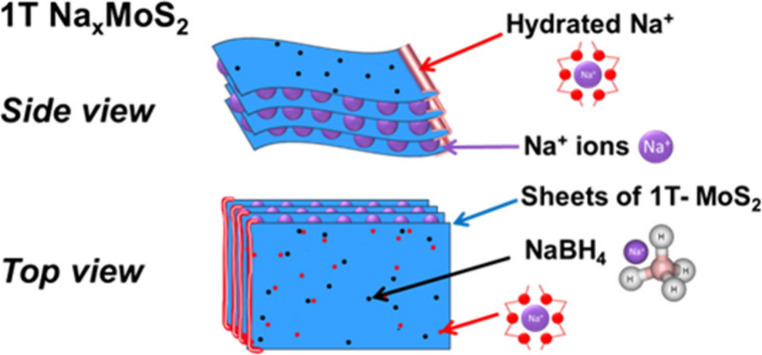

Molybdenum disulfide (MoS_2_) has attracted interest for energy storage and catalysis owing to its layered structure and intrinsic catalytic activity.^1–4^ In its natural form, MoS_2_ adopts the semiconducting 2H polymorph with hexagonal symmetry and trigonal prismatic Mo coordination. The metallic 1T polymorph with octahedral coordination^1,5^ can also be synthesized directly or via phase transformation of the 2H phase. The conventional approach to phase transformation involves the intercalation of alkali metal ions into 2H MoS_2_.^1,5–7^ Such intercalation induces electron transfer and lattice distortion in 2H MoS_2_, which alters the Mo–S coordination environment and drives phase transition from the semiconducting 2H phase to the metallic 1T phase. For example, both Li_*x*_MoS_2_ and nonlithiated 1T MoS_2_ synthesized via chemical and electrochemical methods have been extensively studied and demonstrated in various energy-related applications, including lithium–sulfur (Li–S) batteries and the hydrogen evolution reaction (HER).^1,2,8–11^ The good performance of these materials is primarily attributed to their intrinsic properties, such as high conductivity, catalytic activity, and large surface-to-volume ratio.^1,2,8,12^

Although lithium intercalation in MoS_2_ has been widely explored,^13–23^ the intercalation of sodium is inherently challenging. However, sodium-based intercalation for phase transformation could provide a cost-effective and environmentally friendly pathway for energy applications, particularly for large-scale sodium ion (Na^+^ ion) energy storage systems. For this reason, several studies have reported different methods for the sodiation of 2H MoS_2_ to obtain metallic 1T MoS_2_.^6,24–27^ For example, electrochemical sodiation has been employed to intercalate Na^+^ ions into MoS_2_. This process exploits the weak van der Waals interactions between layers, allowing sodium to be accommodated.^14,25,26,28–34^ However, we have found that in solvent-based synthesis methods, the degree of sodium intercalation is limited, which leads to incomplete phase transformation.^35^ Here, we describe a solid-state reaction between 2H MoS_2_ and sodium borohydride (NaBH_4_) for the production of sodiated 1T phase MoS_2_ (Na_*x*_MoS_2_).^8,17,36–39^ Through optimized synthesis conditions, we obtain a stable phase of 1T Na_*x*_MoS_2_ that is mostly free of borohydride residue, rendering the material highly conductive so that electrodes for Na–S batteries can be fabricated and tested.

## Results and Discussion

### Synthesis Strategy

Sodiation of 2H MoS_2_ was carried out by grinding pre-exfoliated MoS_2_ with sodium borohydride (NaBH_4_) (see Experimental Methods).^8^ The mixture was then annealed at 300 °C for 72 h under argon to achieve sodiation, according to the reaction

$${\mathrm{MoS}}_{2}+{\mathrm{NaBH}}_{4}\to {\mathrm{Na}}_{x}{\mathrm{MoS}}_{2}+\frac{1}{2}B_{2}H_{6}+\frac{1}{2}H_{2}$$

The as-prepared sodiated metallic molybdenum disulfide (1T Na_*x*_MoS_2_) was subsequently washed with dimethylformamide (DMF) to remove residual byproducts (Figure 1).

**Figure 1:**
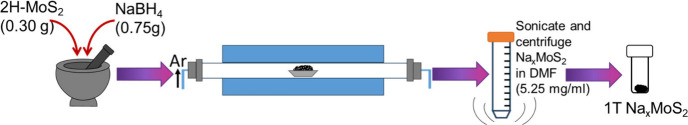
Schematic overview of chemically sodiated 1T MoS_2._.

In this process, the borohydride anion (BH_4_^–^) serves as the reducing agent. Upon heating, NaBH_4_ decomposes to release borane (BH_3_) and hydrogen gas (H_2_), which readily dimerizes at 300 °C to form B_2_H_6_, along with H_2_.^40–42^ The anions, BH_4_^–^, donate electrons that partially reduce Mo^4+^ and drive the insertion of Na^+^ ions between the layers. The combined effects of electron transfer and cation intercalation destabilize the trigonal prismatic coordination of 2H MoS_2_, thereby inducing a phase transition to the metallic octahedral 1T phase.

This strategy was inspired by the LiBH_4_-based method for synthesizing 1T Li_*x*_MoS_2_.^8,17,36–39^ lithium borohydride (LiBH_4_) is a strong reducing agent that enables rapid phase transformation at relatively low temperatures. However, its high reactivity often leads to the over-reduction of Mo–S bonds, which can severely disrupt the layered structure.^39^ Furthermore, LiBH_4_ is extremely sensitive to moisture and oxygen, which limits reproducibility and scalability. To address and mitigate these challenges, we explored the use of NaBH_4_ as an alternative. Its milder reducing capability allows for a more controlled reduction-intercalation process, which enables uniform Na^+^ ion insertion with minimal lattice damage. The larger ionic radius of Na^+^ ion relative to that of Li^+^ ion further promotes interlayer expansion, which stabilizes the 1T phase. In addition, NaBH_4_ is more cost-effective and suitable for gram to kilogram-scale manufacture. Despite these advantages, the relatively larger ionic radius of the Na^+^ ion can hinder diffusion between the layers of 2H MoS_2_. To overcome this limitation, we utilize a pre-exfoliation step to increase the interlayer spacing and generate accessible pathways for Na^+^ ion transport (Supporting Information, Figure S1a). As a result, Na^+^ ions are inserted homogeneously, and a uniform phase transformation is achieved. The combination of pre-exfoliation and NaBH_4_-mediated sodiation yields stable 1T Na_*x*_MoS_2_ for potential large-scale production.

### Phase Identification and Chemical Composition of Na_*x*_MoS_2_

The X-ray diffraction (XRD) patterns of three samples, including unmodified 2H MoS_2_, chemically sodiated MoS_2_ produced by annealing NaBH_4_ with MoS_2_ (CS Na_*x*_MoS_2_), and electrochemically sodiated MoS_2_ prepared by electrochemical intercalation (EC Na_*x*_MoS_2_, see Experimental Methods), are shown in Figure 2a. The XRD pattern of CS Na_*x*_MoS_2_ exhibits an expanded interlayer spacing of the (001) planes, indicated by a peak at 2θ = 12.4°. This reflection corresponds to the (002) plane of layered MoS_2_, which represents the periodicity along the *c*-axis. The shift of this peak from its original position at 14.4° in pristine 2H MoS_2_ indicates an expansion of the interlayer spacing from 0.62 to 0.71 nm, as calculated using Bragg’s law. Such expansion confirms the successful sodium intercalation into the van der Waals gaps between MoS_2_ layers, consistent with previous reports.^24,31,33^ In some batches, additional peaks at lower angles (2θ = 9.5° and 7.5°) were observed. These features likely reflect the variation in interlayer spacings induced by Na intercalation and are reported here for completeness. (Supporting Information, Figure S1b). The reflection at 2θ = 29.2° could indicate the presence of metallic sodium in both the EC and CS Na_*x*_MoS_2_ samples. In EC Na_*x*_MoS_2_, it could originate from residual sodium from the chip, while in CS Na_*x*_MoS_2_ it could come from either surface-contaminated NaBH_4_ or residual metallic sodium.^43,44^

**Figure 2:**
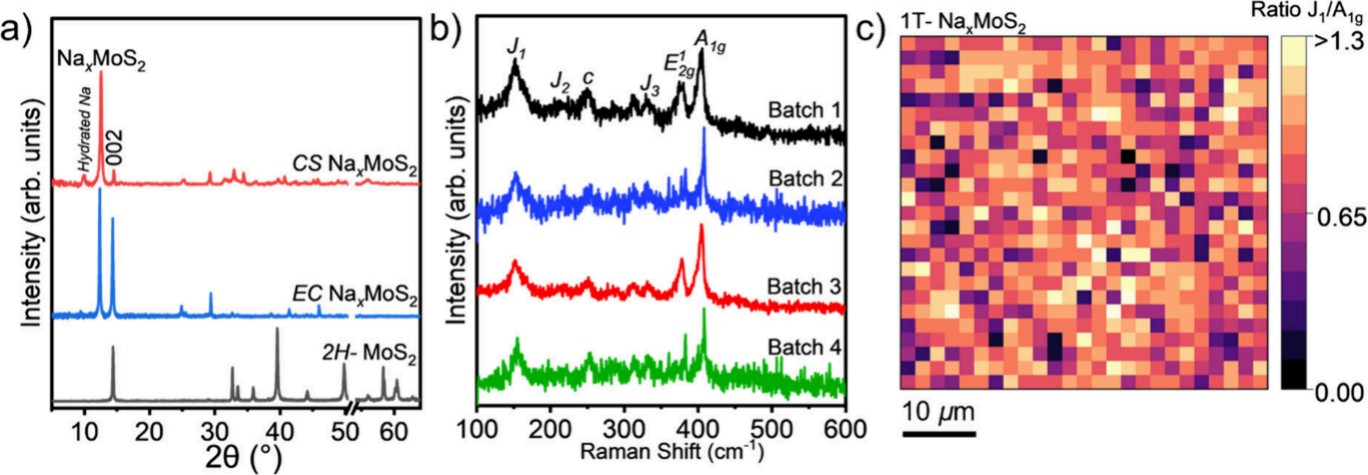
(a) XRD patterns of 1T Na_*x*_MoS_2_ obtained by chemical sodiation (CS) via solid-state reaction with NaBH_4_ and electrochemically (EC) intercalated Na_*x*_MoS_2_, showing a peak at 2θ = 12.4° corresponding to expanded interlayer spacing from sodium intercalation. (b) Raman spectra of chemically sodiated 1T Na_*x*_MoS_2_, highlighting the emergence of the J_1_ peak (along with the J_2_, J_3_, and c peaks),^45^ characteristic of the 1T phase, consistently observed across multiple batches. (c) Raman intensity ratio map (J_1_:A_1g_) over a 50 μm × 50 μm area with 2 μm resolution for Batch 1. The predominance of light orange regions indicates a ∼1:1 peak ratio, demonstrating uniform and extensive conversion to the metallic 1T phase.

Raman spectroscopy was used to monitor the structural transition from the semiconducting 2H phase to the metallic 1T phase of MoS_2_. The appearance of J peaks in the Raman spectra (Figure 2b), consistently observed across multiple batches, confirms the presence of the 1T phase. To assess the spatial uniformity, Raman mapping was conducted over a 50 μm × 50 μm region with a step size of 2 μm (see Experimental Methods). The relative intensity ratios of the J_1_ peaks (characteristic of the 1T phase) to A_1g_ peaks (corresponding to sulfur atoms moving in opposite directions perpendicular to the MoS_2_ plane) were used to monitor the uniformity of the phase transition. The resulting map of the ratios (Figure 2c) was found to be dominated by light orange regions, with scattered purple (weak J_1_ signal) and black (2H phase) areas, indicating that the 1T phase is the prevailing component.

Sodium magic-angle-spinning solid-state nuclear magnetic resonance (^23^Na ssNMR) spectroscopy was used to probe the sodium environment in 1T Na_*x*_MoS_2_ (Figure 3a). The full spectrum, together with the original expanded regions from 250 to −250 ppm and from 75 to −75 ppm, are shown in Figure S2. Both the NaBH_4_ reference (blue) and CS Na_*x*_MoS_2_ product (red) resonances were analyzed to identify the sodium species intercalated within the layered 1T structure. In the NaBH_4_ reference, a sharp resonance appears at −8.66 ppm, corresponding to bulk NaBH_4_.^46^ At 5.50 ppm, a weaker and broader resonance is observed, which corresponds to hydrated NaBH_4_. This assignment is supported by thermogravimetric analysis (TGA, Figure S3), which shows an 8.0% mass loss in the NaBH_4_ granules below 200 °C. This likely comes from exposure of the samples to the atmosphere. The presence of hydrated species also likely accounts for the additional XRD peaks observed at 2θ = 9.5° and 7.5° (Supporting Information, Figure S1b). These peaks correspond to a small fraction of the hydrated sodium species within the interlayers of 1T MoS_2_ (Figure 3b).

**Figure 3:**
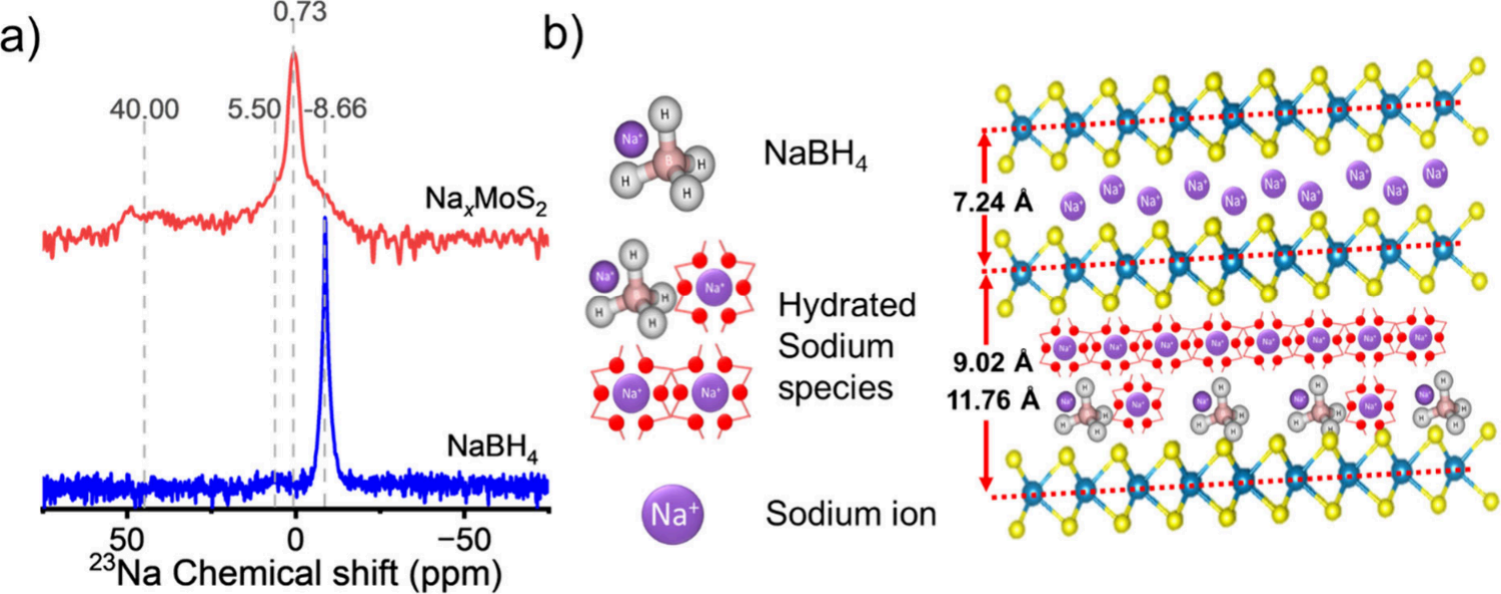
(a) Solid-state ^23^Na MAS NMR spectra of NaBH_4_ (blue) and chemically synthesized Na_*x*_MoS_2_ (red) on a log scale to highlight peak position (original data are shown in the Supporting Information, Figure S2). The broad base of the resonance in Na_*x*_MoS_2_ reflects multiple Na environments arising from intercalation within the 1T MoS_2_ lattice. (b) Schematic of three Na-containing species, Na^+^ ions, hydrated sodium species, and NaBH_4_, each shown to be intercalated within the 1T MoS_2_ layers. The sizes of these species correlate with the degree of lattice expansion, consistent with the low-angle XRD reflections at 7.5° and 9.5° (Figure 2a).

A sharp resonance at 0.73 ppm, characteristic of intercalated Na^+^ ions, is observed in Na_*x*_MoS_2_.^24^ The broadening at the base of the CS Na_*x*_MoS_2_ peak, together with the partial overlap with NaBH_4_ signals at −8.66 and ∼5.50 ppm, suggests trace residual species that can be attributed to unreacted NaBH_4_, surface-bound NaBH_4_, or hydrated sodium species. Despite thorough washing, minor residues persist, but their quantity is negligible (see the Supporting Information, Figures S4 and S5). Additionally, a broad resonance centered near 40 ppm was observed for CS Na_*x*_MoS_2_, which is also likely due to sodium intercalation. This is consistent with chemical shifts for Na^+^-intercalated hard carbons.^47–49^ Together, these findings provide evidence for the successful intercalation of sodium ions and elucidate the distinct local sodium environments within 1T Na_*x*_MoS_2_.

To correlate the spectroscopic measurements with atomic structure, high resolution transmission electron microscopy (HR-TEM) was employed to directly visualize the structure of the synthesized material at the atomic scale. HR-TEM images (Figure 4a) acquired along the (001) plane of MoS_2_ were quantitatively analyzed to estimate the relative fractions of 1T and 2H phases. These finding were cross-validated with XPS mapping to ensure consistency between the surface chemistry and lattice structure. Analysis of several randomly selected areas revealed that up to ∼60% of the material exists in the 1T phase (additional regions are provided in the Supporting Information, Figure S6). The 2H phase displayed a hexagonal arrangement with clearly resolved Mo and S columns, while the 1T phase exhibited octahedrally coordinated Mo atoms with reduced contrast from the single S atoms (Figure 4b). Distinct lattice fringes of 0.32 nm for 2H MoS_2_ and 0.28 nm for 1T MoS_2_ provide further confirmation of phase identification (Figure 4c).

**Figure 4:**
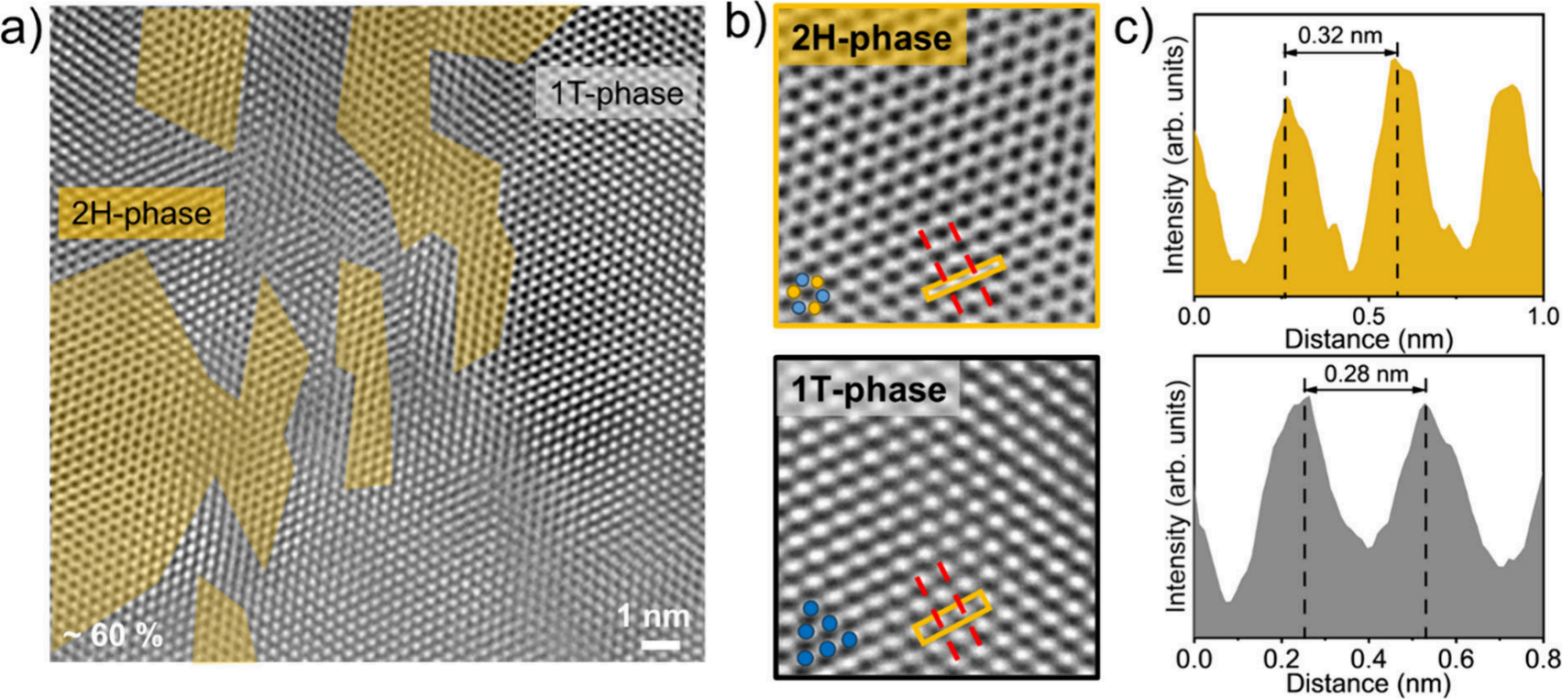
High resolution transmission electron microscopy (HR-TEM) analysis of chemically sodiated 1T MoS_2_. (a) HR-TEM image along the (001) zone axis used to assess the structural phases. Quantitative analysis of five regions indicates ∼40–60% 1T phase content, consistent with XPS mapping. (b) Atomic-resolution images distinguish the semiconducting 2H phase from the metallic 1T phase. Blue spheres mark Mo sites, and yellow spheres denote S columns. (c) Lattice fringes of 2H MoS_2_ (0.32 nm) and 1T MoS_2_ (0.28 nm), confirming phase identification.

X-ray photoelectron spectroscopy (XPS) mapping was performed to quantify the spatial distribution of the 1T phase over macroscopic (1.6 mm × 1.6 mm) regions. Figure 5a shows the mapped area together with a schematic of the measurement grid. Representative Mo 3d spectra from the left and right sides of the sample are displayed in Figure 5b,c, respectively. On the left edge (Figure 5b), the spectra reveal variation in 1T phase content, ranging from 52 to 67% 1T phase. In contrast, spectra from the right edge exhibit a slight positive shift in the Mo 3d_5/2_ binding energy. This shift suggests partial degradation of the material and the formation of metallic Mo at the edges. Such behavior is consistent with previous observations in chemically intercalated Li_*x*_MoS_2_ systems subjected to over-lithiation.^50^ In contrast, spectra collected from the center of the sample (Figure 5d) exhibited high uniformity, with the 1T phase consistently accounting for 56–58% of the signal with a standard deviation of ±9% across the mapped area. Mo 3d spectra from positions 1 and 4 were deconvoluted (additional spectra provided in the Supporting Information, Figure S7a–d) to determine the relative contributions of the 1T and 2H phases within the central mapped column. The analysis confirmed a uniform 1T phase composition in the central region. Overall, spectra from the central area revealed a consistent 1T phase content (56–58%) without evidence of metallic Mo. By contrast, spectra from the edges (Figure 5b,c) displayed deviations including variations in yield, binding energy shifts, and signatures of Mo^0^. This comparison highlights structural uniformity and stability of the bulk material while indicating that edge sites are prone to react during sodium intercalation. Additional XPS spectra for the Na 1s, B 1s, and S 2p regions are provided in the Supporting Information (Figure S4, respectively).

**Figure 5:**
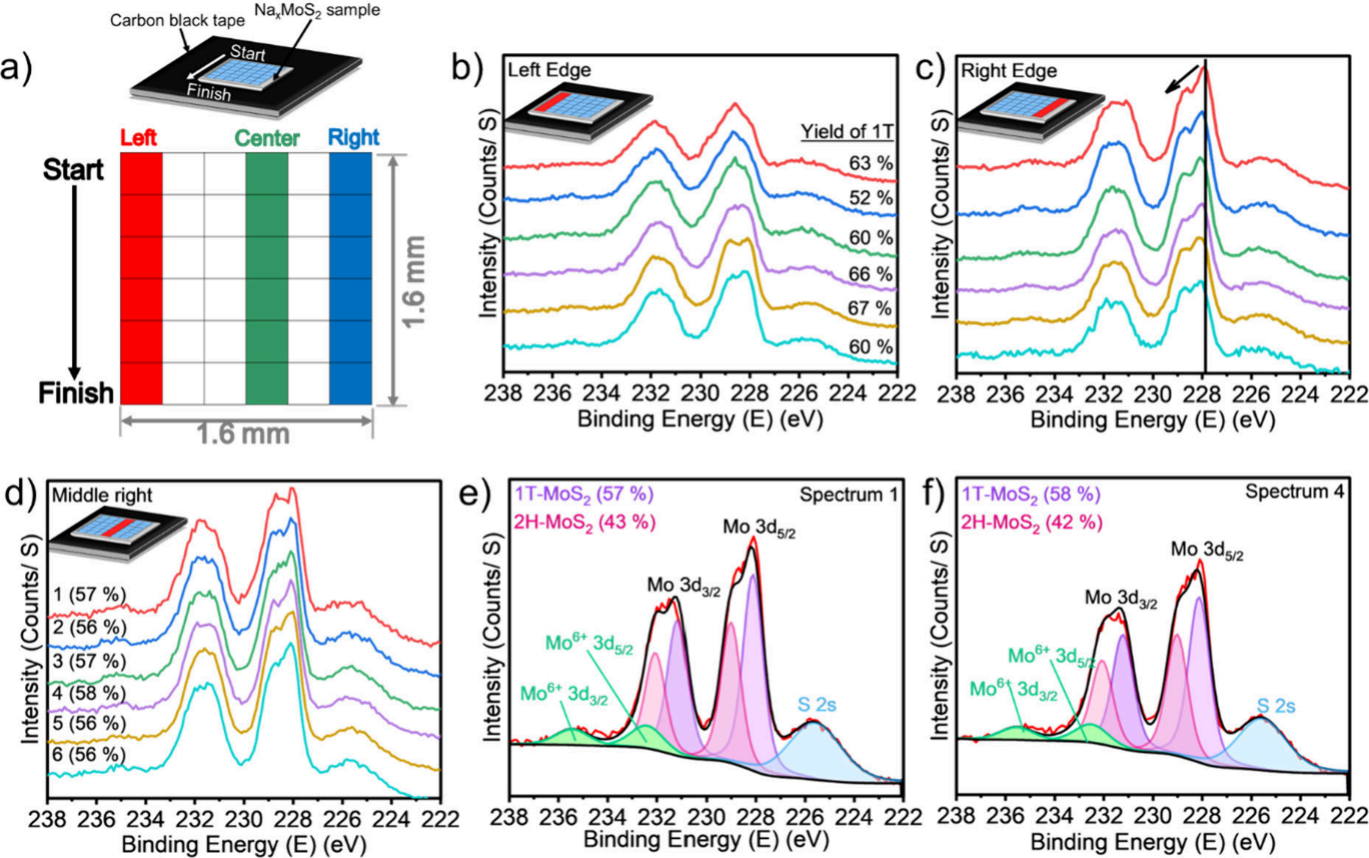
X-ray photoelectron spectroscopy (XPS) mapping of chemically sodiated 1T MoS_2_. (a) Schematic image of the 1.6 mm × 1.6 mm mapped region. (b) Mo 3d spectra collected from the left edge of the sample, showing variation in yield. (c) Mo 3d spectra from the right edge, showing a slight positive shift in the Mo 3d_5/2_ binding energy. The black vertical line shows the Mo 3d_5/2_ peak position, and the arrow indicates the shift toward higher binding energy. (d) Spectra from the central column of the mapped area, exhibiting consistent features across the region. (e, f) Representative Mo 3d_3/5_ spectra from individual points along the central column, with typical peak fittings used to quantify the 1T and 2H phase fractions. Across all spectra (see Figure S7a–d for additional data), the 1T phase consistently accounts for ∼56–58% of the signal.

### Morphology and Elemental Distribution

Scanning electron microscopy (SEM) imaging and energy-dispersive X-ray spectroscopy (EDX) were employed to examine the 3D morphology and elemental distribution of the chemically sodiated 1T MoS_2_. Figure 6a,b displays a uniform layered morphology across different regions and magnifications (additional images are shown in Figure S9). At higher magnification (Figure 6b), interlayer spacing and partially delaminated sheets are evident, providing direct evidence of the structural disruption induced by the chemical sodiation. Cross-sectional TEM analysis further reveals stacked MoS_2_ layers with visibly expanded (001) spacings (Figure 6c). Pristine 2H MoS_2_ typically exhibits an interlayer spacing of 0.61 nm, while Na_*x*_MoS_2_ shows an interlayer spacing of 0.80 nm, supporting the expansion of layers due to sodium intercalation. Elemental mapping by EDX shows the uniform presence of Mo and S across the sample (Figure 6d and Figure S10). Sodium is also homogeneously distributed, whereas no boron signal was detected. To address the inherently low detectability of boron in EDX, we analyzed an unwashed 1T Na_*x*_MoS_2_ sample (Supporting Information, Figure S11). In this case, boron was clearly detected, confirming that its absence in the DMF-washed samples arose from effective removal. This result suggests that NaBH_4_ was effectively removed by DMF washing, leaving only the intercalated sodium ions. Oxygen is present throughout the sample, likely originating from ambient exposure during handling and from hydrated sodium species formed by the atmospheric interaction of NaBH_4_ and Na_*x*_MoS_2_. The sodium content in CS Na_*x*_MoS_2_ was estimated by electrochemical deintercalation measurements (Supporting Information, Figure S12 and eqs S1–S3), which yielded an approximate composition of Na_0.6_MoS_2_. Overall, the morphology of Na_*x*_MoS_2_ is consistent with the exfoliated 1T phase MoS_2_, showing expanded interlayer spacing and partially delaminated sheets. Such features increase the surface area and porosity, thereby facilitating ion transport in electrochemical applications.

**Figure 6:**
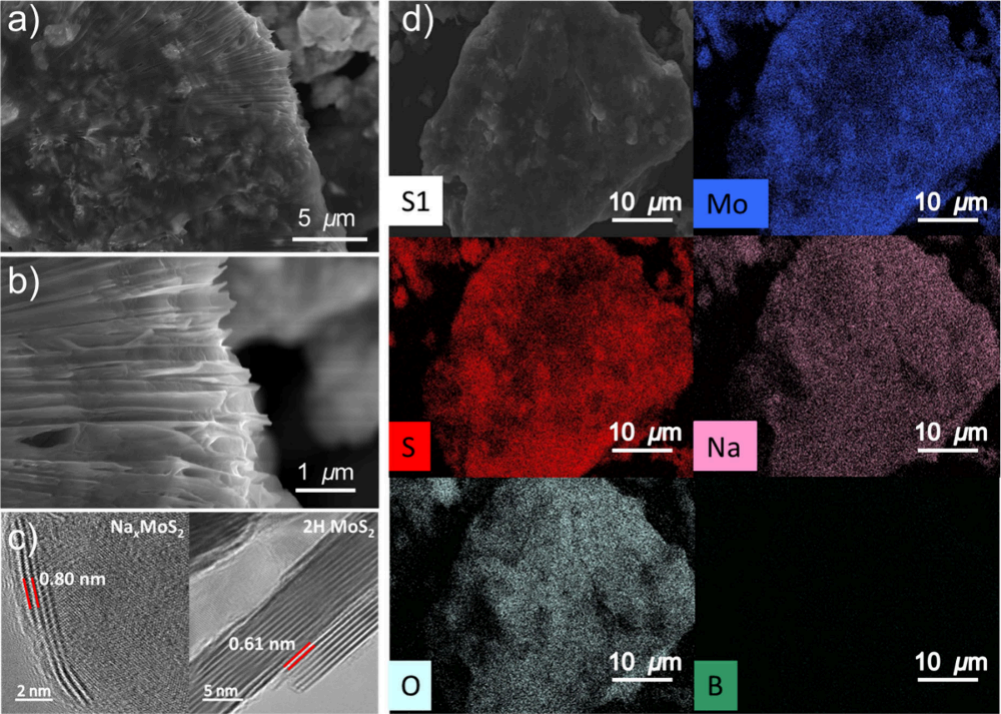
SEM and TEM analyses of chemically sodiated 1T MoS_2_. (a) Low-magnification SEM images showing a stacked, layered morphology. (b) Higher-magnification image highlighting interlayer voids and sheets. (c) Cross-sectional TEM images of expanded interlayer spacing for chemically sodiated 1T MoS_2_ (0.80 nm) and pristine 2H MoS_2_ (0.61 nm). (d) Top-view SEM image of sodiated 1T MoS_2_ with corresponding EDX elemental maps of Mo (blue), S (red), Na (pink), O (light blue), and B (green). Mo and S signals confirm the uniform layered distribution, while Na is consistently detected throughout the sample. The O signal is attributed to atmospheric exposure.

### Electrochemical Performance of Na_*x*_MoS_2_ as Sulfur Host in Na–S Batteries

Our recent work on lithiated MoS_2_ (Li_*x*_MoS_2_) as a sulfur cathode host showed that prelithiation facilitates Li diffusion, which enhances sulfur utilization in lithium–sulfur batteries. We therefore tested the efficacy of Na_*x*_MoS_2_ as a sulfur host for room temperature (RT) Na–S batteries. While high temperature Na–S batteries have been utilized for decades,^51,52^ the poor kinetics in Na–S batteries have limited their use at RT. Na_*x*_MoS_2_/sulfur (Na_*x*_MoS_2_/S) cathodes were prepared by the conventional slurry-casting method (see Experimental Methods). The cathode morphology was examined by SEM (Figure S13). Additional characterization by XRD and Raman spectroscopy before and after cycling confirmed the uniform morphology and structural stability of the prepared cathodes (Figure S14). Properties of RT Na–S coin cell batteries with the Na_*x*_MoS_2_/S, carbon/sulfur (C/S), and 2H MoS_2_/sulfur (2H MoS_2_/S) cathodes are shown in Figure 7a–c, respectively. The Na_*x*_MoS_2_/S cathodes exhibited a higher specific capacity (1029 mAh g^–1^) than the C/S (222 mAh g^–1^) and 2H MoS_2_/S (170 mAh g^–1^) electrodes. While the discharge profiles are consistent with those reported in the literature, the characteristic plateaus are less pronounced.^53^ This suggests that further optimization, particularly in electrolyte formulation and electrode–electrolyte pairing, will be necessary to fully exploit the capabilities of the Na_*x*_MoS_2_ material as a S host for RT Na–S batteries. Carbonate-based electrolytes were not further pursued in this study, as they are generally less compatible with polysulfide chemistry in room temperature Na–S systems and may suppress reversible sulfur redox behavior. Accordingly, 1.0 M NaCF_3_SO_3_ in TEGDME was employed as a more suitable electrolyte for probing the fundamental electrochemical behavior of the system. However, a gradual capacity loss between the first and tenth cycles was observed, implying the partial loss of active material to the electrolyte.^53–58^ Nevertheless, the Na_*x*_MoS_2_/S delivered an ∼4 times higher capacity than the C/S and 2H MoS_2_/S cathodes, underscoring the effectiveness of Na_*x*_MoS_2_ as a sulfur host.

**Figure 7:**
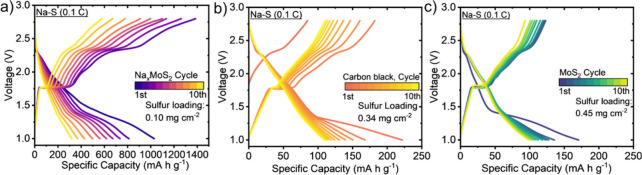
Galvanic charge–discharge curves of (a) Na_*x*_MoS_2_/S, (b) C/S, and (c) 2H MoS_2_/S cathodes in the same Na–S battery system at 0.1 C, showing the higher specific capacity for Na_*x*_MoS_2_.

To further isolate the intrinsic behavior of the cathode, the same material was evaluated in a Li–S battery configuration, where anode stability and electrolyte chemistry are more established. As shown in the Supporting Information (Figure S15a), the Coulombic efficiency and specific capacity were evaluated at different C-rates. While the Na–S response is modest, the Li–S configuration delivers high specific capacity at multiple C-rates. The cathode exhibits stable electrochemical behavior, with capacity decreasing from 1079 to 929 mAh g^–1^ over 10 cycles at 0.1 C (Figure S15b). These results provide supporting evidence for the intrinsic characteristics of the cathode and suggest that further optimization of the electrolyte composition and electrode architecture will be important to fully exploit its potential in RT Na–S batteries.

Differential capacity analysis of the first cycle in Na–S batteries shown in Figure 8a was used to obtain insights into the catalytic role of Na_*x*_MoS_2_. The charge peak for the Na_*x*_MoS_2_ host occurs at 20 mV lower than in the carbon host electrode. Furthermore, the peak at 2.35 V is more prominent in the Na_*x*_MoS_2_ cathode host coin cells. This behavior indicates slightly enhanced catalytic activity for the charging conversion of the sodium polysulfides (NaPSs). Similarly, the higher discharge peak at 1.35 V, compared to that of the C/S cathodes, suggests that the sulfur reduction reaction (SRR) to Na_2_S is more effectively facilitated by Na_*x*_MoS_2_.^53^

**Figure 8:**
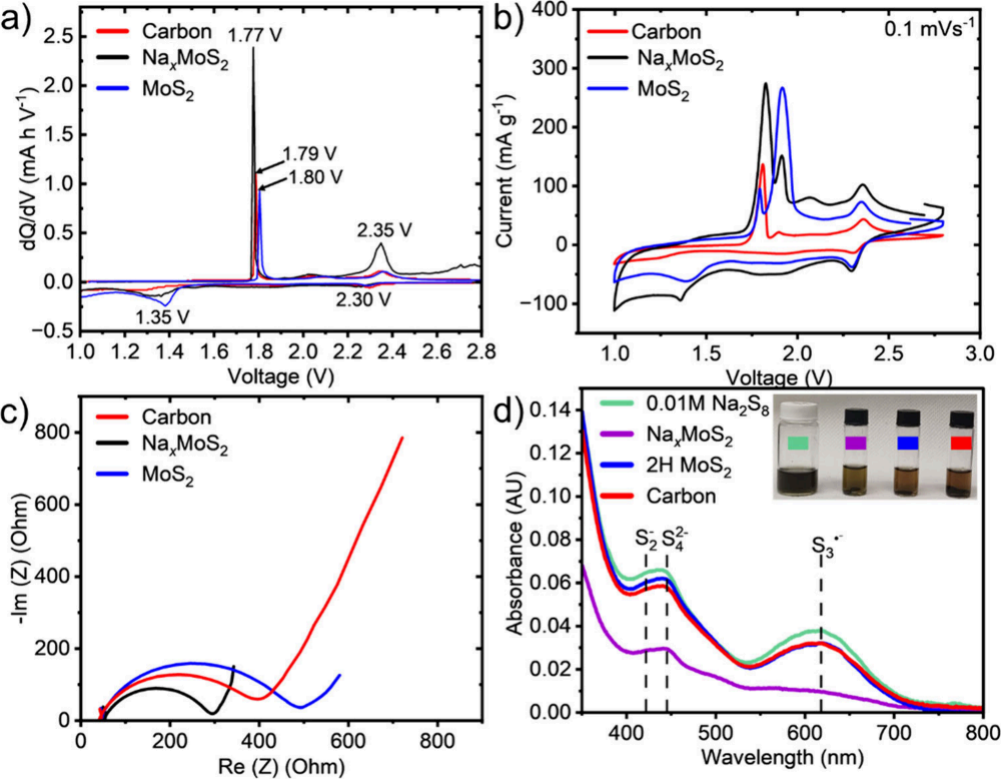
Electrochemical and catalytic comparison of Na_*x*_MoS_2_/S (black and purple), C/S (red), and 2H MoS_2_/S (blue) cathodes in room temperature Na–S batteries using 1.0 M NaCF_3_SO_3_ in TEGDME electrolyte. (a) Differential capacity curves of the first cycle. (b) Cyclic voltammetry (CV) profiles of all electrodes at a 0.1 mV s^–1^ rate. (c) Electrochemical impedance spectra (EIS) before cycling, showing the lowest charge-transfer resistance for Na_*x*_MoS_2_/S and the highest for 2H MoS_2_/S. (d) UV–vis absorbance spectra of 0.01 M Na_2_S_8_ in TEGDME electrolyte before and after exposure to each cathode, demonstrating the effective suppression of the S_3_^•–^ radical by Na_*x*_MoS_2_/S.

Cyclic voltammetry (CV) measurements (Figure 8b) further support these observations. Compared to C/S and 2H MoS_2_/S electrodes, the Na_*x*_MoS_2_/S electrode exhibits higher current densities for both oxidation and reduction peaks, suggesting more favorable sulfur redox kinetics. Notably, the primary oxidation peak associated with Na_2_S to higher-order polysulfides appears at a lower potential (∼1.80 V) for Na_*x*_MoS_2_, whereas for 2H MoS_2_ this oxidation peak is at a higher potential (∼1.90 V).^53^ This reduced overpotential indicates a more efficient adsorption and conversion of polysulfide intermediates, which may help limit active material loss within the electrolyte.^53^ Additionally, the oxidation region (1.80 to 2.10 V) shows a multipeak feature for Na_*x*_MoS_2_/S and MoS_2_/S electrodes, in contrast to the single and less defined peak observed for the carbon control, suggesting stepwise polysulfide oxidation. These features are attributed to the sequential conversion of polysulfides into higher-order species and ultimately to S_8_.^54^ The differences in peak intensity reflect variations in catalytic activity and reaction kinetics among the samples. Notably, the Na_*x*_MoS_2_/S electrode shows more enhanced oxidation peak features associated with the conversion of lower-order polysulfides into longer-chain species, indicating improved catalytic facilitation of polysulfide transformation.

Electrochemical impedance spectroscopy (EIS) performed prior to cycling (Figure 8c) provides further insight. The Na_*x*_MoS_2_/S cathode exhibits the lowest charge-transfer resistance among the three systems, indicating more efficient interfacial charge transport and reaction kinetics. In contrast, the higher resistance observed for 2H MoS_2_ is consistent with its weaker interaction with polysulfide intermediates and slower redox conversion.

This enhanced polysulfide adsorption is directly reflected in the ultraviolet–visible (UV–vis) absorption spectra (Figure 8d). The 0.01 M Na_2_S_8_ solution in 1.0 M NaCF_3_SO_3_ in TEGDME exposed Na_*x*_MoS_2_ shows a clear suppression of the characteristic S_3_^•–^ radical adsorption compared to carbon and 2H MoS_2_, confirming stronger polysulfide adsorption and confinement. Collectively, the CV, EIS, and UV–vis results support the role of 1T Na_*x*_MoS_2_ in promoting sulfur redox kinetics, reducing interfacial resistance, and limiting active material loss during electrochemical cycling.

Taken together, these results suggest that the 1T Na_*x*_MoS_2_ host enhances capacity by the enhanced diffusion and intercalation of sodium ions. It also increases the conductivity so that efficient electron transfer during redox reactions can occur. Sodium intercalation in 1T Na_*x*_MoS_2_ also expands the interlayer spacing, which improves accessibility for polysulfides and stabilizes the layered framework. Catalytically active Mo sites accelerate the conversion of NaPSs into Na_2_S, while defect-rich regions in the layered structure help to anchor soluble species. Through this combination of conductivity, structural stability, catalytic activity, and polysulfide confinement, 1T Na_*x*_MoS_2_ stabilizes the sulfur cathode and delivers substantially enhanced capacity in RT Na–S batteries. To fully realize the potential of Na_*x*_MoS_2_, further optimization, particularly under high-sulfur-loading conditions, together with density functional theory calculations and electrolyte refinement, will be important for advancing toward practical next-generation sulfur-based battery systems.

## Conclusions

This study describes a solid-state chemical sodiation method using sodium borohydride for the 2H to 1T phase conversion of MoS_2_, achieving ∼60% phase conversion. We also describe a method of repeated DMF washing to remove borohydride residue to achieve 1T phase Na_*x*_MoS_2_. Structural and compositional characterization by XRD, Raman mapping, TEM, XPS mapping, NMR, SEM, and EDX confirm the sodium incorporation, uniform 1T phase distribution, and structural stability. Electrochemical testing shows that Na_*x*_MoS_2_/S cathodes are promising for RT Na–S batteries compared to the conventional C/S and the 2H MoS_2_/S cathodes, delivering reasonable capacity and cyclability. Future optimization of the electrolyte composition and electrode design, as well as density functional theory calculations, will be essential to fully exploit its advantages. Even so, the present results suggest that chemically sodiated 1T MoS_2_ is a compelling platform that connects intercalation science with the design of next-generation rechargeable batteries.

## Experimental Methods

### Chemical Synthesis of Sodiated 1T MoS_2_

2H MoS_2_ (<2 μm particle size, purchased by Sigma-Aldrich) was pre-exfoliated in *N*-methyl-2-pyrrolidone (NMP, 60 mL, 99%, Merck), sonicated in an ice bath for 1 h, and centrifuged (15 min, 4500 rpm) to collect the solid. This was dried overnight in a vacuum oven (60 °C, 12 h).

For chemical exfoliation, 2H MoS_2_ (0.30 g) was ground with NaBH_4_ (0.75 g, granules, Prill, ≥99%, Honeywell Fluka) inside an Ar-filled glovebox (H_2_O and O_2_ < 0.1 ppm). The mixture was transferred to a tube furnace, gradually heated to 120 °C at a ramping rate of 5 °C min^–1^, and held for 1 h to then heat to 300 °C for 72 h with a ramping rate 5 °C min^–1^ under flowing argon before natural cooling.

The resultant product was purified by sequential washing: three times with dimethylformamide (DMF) (200 mL, anhydrous 99.8%, Sigma-Aldrich), sonicating each wash for 1 h in an ice bath. The sample was centrifuged at 4500 rpm for 15 min after each sonication to collect the solids. The solid was dried under vacuum at room temperature.

### Electrochemically Sodiated 1T MoS_2_

The cathode was prepared by ball milling a 80:10:10 mass ratio of 2H MoS_2_, carbon (Super P), and polyvinylidene fluoride (PVDF) in NMP. The ball milling was conducted in a THINKY Mixer (THINKY ARV-501). The slurry was doctor-bladed onto copper foil (150 μm thickness), dried for 24 h at 60 °C under vacuum, and punched into 12 mm diameter electrodes. The MoS_2_ electrodes were assembled in coin cells (CR2025, Cambridge Energy Solutions) in an argon-filled glovebox (O_2_ < 0.5 ppm, H_2_O < 0.5 ppm). The assembly included the 2H MoS_2_ electrode, a 16 mm diameter sodium chip (Landt Instruments, Battery Test Equipment & Supplies), two polypropylene separators (Celgard), and an electrolyte (1 M NaPF_6_ in EC:DEC (1/1) with 10 wt % FEC). The cell was rested for 20 h, and galvanostatic charge–discharge (GCD) measurements were performed on a battery cycler (LANDT CT3002A, 1U) in the voltage range of 2.60 to 0.65 V at a current density of 0.02 C (1 C = 670 mAh g^–1^, to the mass of 2H MoS_2_).

### Material Characterization

X-ray diffraction (XRD) was performed using a Bruker D8 Advance Powder Diffractometer with Cu Kα radiation, automatic divergence slits, and a LynxEye-XE position-sensitive detector. Powdered samples were analyzed in silicon holders with a Ø5 mm cavity, and the electrodes were analyzed with an ECC-Opto XRD Beryllium window (0.2 mm thickness), with the electrode facing up. Raman spectroscopy and Raman mapping were conducted using a Horiba LabRAM Odyssey system with a 532 nm laser and a 50× long working distance objective. Mapping was carried out over a 50 μm × 50 μm area with a 2 μm step size. Transmission electron microscopy (TEM) was performed on an FEI Tecnai F20 instrument operating at 200 kV. TEM samples were prepared by drop-casting THF-dispersed powders onto copper grids supported by lacey carbon films and dried under vacuum. X-ray photoelectron spectroscopy (XPS) was conducted using a Thermo Fisher Scientific Nexsa G2 instrument with an Al Kα source; powder samples were washed with deionized water and mounted on carbon tape. Solid-state nuclear magnetic resonance (ssNMR) spectroscopy was performed on samples that were prepared in an argon-filled glovebox (O_2_ < 0.1 ppm, H_2_O < 5 ppm; MBraun). NaBH_4_ (granules, Prill, ≥99%, Honeywell Fluka) was used as a bulk sample and ground to a fine powder before being put into a rotor. Synthetic Na_*x*_MoS_2_ was diluted with dry potassium bromide (KBr). The spectra were recorded using a Bruker Avance III HD Console, with a 16.4 T Bruker wide-bore magnet and using a 1.3 mm Bruker HX MAS probe. A spinning speed of 40 kHz was used. ^23^Na pulses were optimized, and chemical shifts were referenced using NaCl (s) (^23^Na = +7.2 ppm). Scanning electron microscopy (SEM) and energy-dispersive x-ray spectroscopy (EDX) analyses were carried out using an FEI Nova NanoSEM 450 Field Emission Gun Scanning Electron Microscope (FEG-SEM). UV–vis spectra for the cathode absorption tests were collected on a two-channel Lambda 750 spectrometer (PerkinElmer) using argon-filled, sealed quartz glass cuvettes (Hellma Analytics, QS115). Each solution was diluted by a factor of 10.

### Electrochemical Characterization

#### Fabrication of Na–S Cathodes

##### Preparation of MoS_2_/S Composites

Na_*x*_MoS_2_ and 2H MoS_2_ were mixed with sulfur and Super P carbon in a weight ratio of 28:28:44 (MoS_2_:carbon:sulfur). The mixtures were annealed under Ar at 155 °C for 12 h to obtain Na_*x*_MoS_2_/S and 2H MoS_2_/S composites.

##### Slurry Preparation

The as-prepared composites were dispersed in *N*-methyl-2-pyrrolidone (NMP) with polyvinylidene fluoride (PVDF) binder (90:10 wt %) to form a homogeneous slurry. The mixture was homogenized by using a THINKY mixer (1500 rpm for 90 s, followed by defoaming at 100 rpm for 60 s).

##### Electrode Fabrication

The slurry was doctor-bladed (150 μm thickness) onto carbon-coated aluminum foil and dried under vacuum at 60 °C for 24 h. The electrodes were punched into 12 mm diameter disks. Based on this composition, the final sulfur content in the electrode was approximately 40 wt %, calculated from the weight of the electrode.

##### Control Cathode (C/S)

For comparison, C/S cathodes were prepared using sulfur, Super P carbon, and PVDF in a weight ratio of 40:50:10 following the same procedure.

#### Preparation of the Electrolytes and Polysulfide Solution

All electrolytes were prepared by drying the salts overnight in a vacuum oven, followed by transferring the salts and solvents into an Ar-filled glovebox (H_2_O and O_2_ levels below 0.1 ppm). Solutions were prepared via stirring at room temperature for 24 h inside the glovebox. The electrolyte for all Na–S batteries was 1 M sodium trifluoromethanesulfonate (NaCF_3_SO_3_) (Sigma-Aldrich) in tetraethylene glycol dimethyl ether (TEGDME) (Sigma-Aldrich). The 0.01 M Na_2_S_8_ solution was prepared by mixing sodium sulfide (Na_2_S) (Sigma-Aldrich) with nanosulfur (Nanografi Nano Technology) in a stoichiometric ratio in the TEGDME electrolyte.

#### Assembly and Testing of Coin Cells

The electrochemical performance of all the cathodes was evaluated in coin cells (CR2032, Cambridge Energy Solutions), which were assembled in an Ar-filled glovebox. Each cell consisted of 12 mm diameter catalytic host/S as a cathode, a 16 mm cut diameter sodium chip as an anode (Landt Instruments, Battery Test Equipment & Supplies), a 19 mm cut diameter glass fiber (Whatman GFD) separator, and the electrolyte as prepared as previously stated. The separator was fully wetted with the electrolyte overflooded during assembly. The electrochemical measurements for coin cells were conducted on a battery cycler (LANDT CT3002A, 1U). Galvanostatic charge–discharge (GCD) tests were performed in the voltage range of 2.8 to 1.0 V at a rate of 0.1 C (1 C = 1672 mAh g^–1^). Cyclic voltammetry (CV) measurements were performed within a voltage window of 2.8 to 1.0 V at a scan rate of 0.1 mV s^–1^. Electrochemical impedance spectroscopy (EIS) was conducted prior to cycling at open-circuit potential with an amplitude of 10 mV over a frequency range spanning 100 kHz to 10 mHz.

## Supplementary Material



## Notes

Additional material characterization data (XRD patterns, ^23^Na MAS NMR spectra, TGA curve, XPS spectra, SEM and EDX mapping images, and HR-TEM images), calculations of the quantification of sodium in 1T Na_*x*_MoS_2_, and additional cathode characterization and battery analysis (PDF)

## References

[1] Voiry D., Yang J., Chhowalla M. (2016). Recent Strategies for Improving the
Catalytic Activity of 2D TMD Nanosheets Toward the Hydrogen Evolution
Reaction. Adv. Mater..

[2] Li Z., Sami I., Yang J., Li J., Kumar R. V., Chhowalla M. (2023). Lithiated Metallic Molybdenum Disulfide
Nanosheets
for High-Performance Lithium-Sulfur Batteries. Nat. Energy..

[3] Hojaji E., Andritsos E. I., Li Z., Chhowalla M., Lekakou C., Cai Q. (2022). DFT Simulation-Based Design of 1T-MoS_2_ Cathode Hosts for Li-S Batteries and Experimental Evaluation. Int. J. Mol. Sci..

[4] Voiry D., Mohite A., Chhowalla M. (2015). Phase Engineering of Transition Metal
Dichalcogenides. Chem. Soc. Rev..

[5] Chhowalla M., Shin H. S., Eda G., Li L.-J., Loh K. P., Zhang H. (2013). The Chemistry of Two-Dimensional
Layered Transition Metal Dichalcogenide
Nanosheets. Nat. Chem..

[6] Somoano R. B., Hadek V., Rembaum A. (1973). Alkali Metal
Intercalates of Molybdenum
Disulfide. J. Chem. Phys..

[7] Whittingham M. S. (1978). Chemistry
of Intercalation Compounds: Metal Guests in Chalcogenide Hosts. Prog. Solid State Chem..

[8] Voiry D., Salehi M., Silva R., Fujita T., Chen M., Asefa T., Shenoy V. B., Eda G., Chhowalla M. (2013). Conducting
MoS_2_ Nanosheets as Catalysts for Hydrogen Evolution Reaction. Nano Lett..

[9] Miremadi B. K., Morrison S. R. (1987). High Activity Catalyst From Exfoliated MoS_2_. J. Catal..

[10] Zeng Z., Yin Z., Huang X., Li H., He Q., Lu G., Boey F., Zhang H. (2011). Single-Layer
Semiconducting Nanosheets:
High-Yield Preparation and Device Fabrication. Angew. Chem., Int. Ed..

[11] Winter M., Besenhard J. O., Spahr M. E., Novák P. (1998). Insertion
Electrode Materials for Rechargeable Lithium Batteries. Adv. Mater..

[12] Gee M. A., Frindt R. F., Joensen P., Morrison S. R. (1986). Inclusion Compounds
of MoS_2_. Mater. Res. Bull..

[13] Sen U. K., Johari P., Basu S., Nayak C., Mitra S. (2014). An Experimental
and Computational Study to Understand the Lithium Storage Mechanism
in Molybdenum Disulfide. Nanoscale.

[14] Zou J., Li F., Bissett M. A., Kim F., Hardwick L. J. (2020). Intercalation Behaviour
of Li and Na into 3-layer and Multilayer MoS_2_ Flakes. Electrochim. Acta.

[15] Whittingham M. S. (1979). Intercalation
Chemistry and Energy Storage. J. Solid State
Chem..

[16] Benavente E., Santa Ana M. A., Mendizábal F., González G. (2002). Intercalation
Chemistry of Molybdenum Disulfide. Coord. Chem.
Rev..

[17] Py M. A., Haering R. R. (1983). Structural
Destabilization Induced by Lithium Intercalation
in MoS_2_ and Related Compounds. Can.
J. Phys..

[18] Wilson J.
A., Yoffe A. D. (1969). The Transition
Metal Dichalcogenides Discussion and
Interpretation of the Observed Optical, Electrical and Structural
Properties. Adv. Phys..

[19] Bissessur R., Kanatzidis M. G., Schindler J. L., Kannewurf C. R. (1993). Encapsulation
of Polymers into MoS_2_ and Metal to Insulator Transition
in Metastable MoS_2_. J. Chem. Soc.,
Chem. Commun..

[20] Frindt R. F., Yoffe A. D. (1963). Physical Properties
of Layer Structures: Optical Properties
and Photoconductivity of Thin Crystals of Molybdenum Disulphide. Proc. R. Soc. A.

[21] Eda G., Yamaguchi H., Voiry D., Fujita T., Chen M., Chhowalla M. (2011). Photoluminescence
from Chemically Exfoliated MoS_2_. Nano Lett..

[22] Eda G., Fujita T., Yamaguchi H., Voiry D., Chen M., Chhowalla M. (2012). Coherent Atomic
and Electronic Heterostructures of
Single-Layer MoS_2_. ACS Nano.

[23] George C., Morris A. J., Modarres M. H., De Volder M. (2016). Structural
Evolution of Electrochemically Lithiated MoS_2_ Nanosheets
and the Role of Carbon Additive in Li-Ion Batteries. Chem. Mater..

[24] Zheng J., Zhang H., Dong S., Liu Y., Tai Nai C., Suk Shin H., Young Jeong H., Liu B., Ping Loh K. (2014). High Yield
Exfoliation of Two-Dimensional Chalcogenides using Sodium Naphthalenide. Nat. Commun..

[25] Zak A., Feldman Y., Lyakhovitskaya V., Leitus G., Popovitz-Biro R., Wachtel E., Cohen H., Reich S., Tenne R. (2002). Alkali Metal
Intercalated Fullerene-Like MS_2_ (M = W, Mo) Nanoparticles
and Their Properties. J. Am. Chem. Soc..

[26] Cen M., Yan R., Luo X., Liu H., Chen B., Zhang S., Peng W., Li Y., Zhang Q., Fan X. (2025). Pre-intercalated
Sodium Ions Enhance Sodium Storage of MoS_2_ Anode by Mitigating
Structural Dissociation. Nano Lett..

[27] Somoano R. B., Rembaum A. (1971). Superconductivity in
Intercalated Molybdenum Disulfide. Phys. Rev.
Lett..

[28] Wang G., Zhang Y., Cho H. S., Zhao X., Kim F., Zou J. (2021). Revisiting the Structural Evolution of MoS_2_ During Alkali
Metal (Li, Na, and K) Intercalation. ACS Appl.
Energy Mater..

[29] Huang Q., Li X., Sun M., Zhang L., Song C., Zhu L., Chen P., Xu Z., Wang W., Bai X. (2017). The Mechanistic
Insights into the 2H-1T Phase Transition of MoS_2_ upon Alkali
Metal Intercalation: From the Study of Dynamic Sodiation Processes
of MoS_2_ Nanosheets. Adv. Mater. Interfaces.

[30] Gao P., Wang L., Zhang Y., Huang Y., Liu K. (2015). Atomic-Scale
Probing of the Dynamics of Sodium Transport and Intercalation-Induced
Phase Transformations in MoS_2_. ACS
Nano.

[31] Wang X., Shen X., Wang Z., Yu R., Chen L. (2014). Atomic-Scale
Clarification of Structural Transition of MoS_2_ Upon Sodium
Intercalation. ACS Nano.

[32] Li Q., Yao Z., Wu J., Mitra S., Hao S., Sahu T. S., Li Y., Wolverton C., Dravid V. P. (2017). Intermediate Phases in Sodium Intercalation
into MoS_2_ Nanosheets and their Implications for Sodium-Ion
Batteries. Nano Energy..

[33] Cook J. B., Ko J. S., Lin T. C., Robertson D. D., Kim H.-S., Yan Y., Yao Y., Dunn B. S., Tolbert S. H. (2023). Ultrafast Sodium Intercalation Pseudocapacitance
in
MoS_2_ Facilitated by Phase Transition Suppression. ACS Appl. Energy Mater..

[34] Li Z., Jiang K., Khan F., Goswami A., Liu J., Passian A., Thundat T. (2019). Anomalous Interfacial Stress Generation
During Sodium Intercalation/Extraction in MoS_2_ Thin-Film
Anodes. Sci. Adv..

[35] Gray E. L., Lee J.-I., Yang Z. J., Wang Y., Chhowalla M. (2025). In Situ Raman
Mapping of Electrochemical Sodium Intercalation and Phase Stability
in MoS_2_ for Sodium-Ion Batteries. ACS Appl. Nano Mater..

[36] Tsai H.-L., Heising J., Schindler J. L., Kannewurf C. R., Kanatzidis M. G. (1997). Exfoliated-Restacked Phase of WS_2_. Chem. Mater..

[37] Heising J., Kanatzidis M. G. (1999). Exfoliated
and Restacked MoS_2_ and WS_2_: Ionic or Neutral
Species? Encapsulation and Ordering of
Hard Electropositive Cations. J. Am. Chem. Soc..

[38] Tsai H.-L., Schindler J. L., Kannewurf C. R., Kanatzidis M. G. (1997). Plastic
Superconducting Polymer-NbSe_2_ Nanocomposites. Chem. Mater..

[39] Zhu J., Wang H., Liu J., Ouyang L., Zhu M. (2017). Exfoliation
of MoS_2_ and h-BN Nanosheets by Hydrolysis of LiBH_4_. Nanotechnology.

[40] Yao Y., Hoffmann R. (2011). BH3 under Pressure: Leaving the Molecular Diborane
Motif. J. Am. Chem. Soc..

[41] Huyen T. L., Huyen T. T. T., Nam C. C., Nam P. C. (2025). Sustainable Hydrogen
Production via CO_2_-assisted BH_3_ + BH_3_ Reaction: a Computational Analysis. RSC Adv..

[42] Sergeev Y. V., Dolinska M. B., Wingfield P. T. (2014). Thermodynamic
Analysis of Weak Protein
Interactions using Sedimentation Equilibrium. Curr. Protoc. Protein Sci..

[43] Zhang B., Ghimbeu C. M., Laberty C., Vix-Guterl C., Tarascon J.-M. (2016). Correlation Between Microstructure and Na Storage Behavior
in Hard Carbon. Adv. Energy Mater..

[44] Rohendi D., Syarif N., Wati E. K. (2019). Storage
and Release of Hydrogen as
a Fuel of the Fuel Cell with Media of NaBO_2_/NaBH_4_. IOP Conf. Ser.: Earth Environ. Sci..

[45] Yang Z. J., Li Z., Lampronti G. I., Lee J.-I., Wang Y., Day J., Chhowalla M. (2024). Environmental
and Thermal Stability of Chemically Exfoliated
Li_x_MoS_2_ for Lithium-Sulfur Batteries. Chem. Mater..

[46] Garroni S., Milanese C., Pottmaier D., Mulas G., Nolis P., Girella A., Caputo R., Olid D., Teixdor F., Baricco M. (2011). Experimental
Evidence of Na_2_[B_12_H_12_] and Na Formation
in the Desorption Pathway of the
2NaBH_4_ + MgH_2_ System. J. Phys. Chem. C.

[47] Stratford J. M., Mayo M., Allan P. K., Pecher O., Borkiewicz O. J., Wiaderek K. M., Chapman K. W., Pickard C. J., Morris A. J., Grey C. P. (2017). Investigating Sodium Storage Mechanisms in Tin Anodes:
A Combined Pair Distribution Function Analysis, Density Functional
Theory, and Solid-State NMR Approach. J. Am.
Chem. Soc..

[48] O’Keefe, C. A. ; Grey, C. P. NMR Investigations of Sodium-Ion Batteries. Sodium-Ion Batteries: Materials, Characterization, and Technology; Wiley, 2022; pp 215–257.

[49] Stratford J. M., Allan P. K., Pecher O., Chater P. A., Grey C. P. (2016). Mechanistic
Insights into Sodium Storage in Hard Carbon Anodes Using Local Structure
Probes. Chem. Commun..

[50] Yang Z. J., Li Z., Loh L., Moloney J., Walmsley J., Li J., Chen Y., Liu L., Zang H., Yan H. (2026). Scalable Manufacture
of Nearly Pure-Phase Metallic MoS_2_ Nanosheets. Nat. Mater..

[51] Oshima T., Kajita M., Okuno A. (2004). Development of Sodium-Sulfur Batteries. Int. J. Appl. Ceram. Technol..

[52] Kumar D., Rajouria S. K., Kuhar S. B., Kanchan D. K. (2017). Progress and Prospects
of Sodium-Sulfur Batteries: A Review. Solid
State Ion..

[53] Gray E. L., Lee J.-I., Li Z., Moloney J., Yang Z. J., Chhowalla M. (2025). Mapping Polysulfides in Sodium-Sulfur Batteries. ACS Nano.

[54] Ryu H., Kim T., Kim K., Ahn J.-H., Nam T., Wang G., Ahn H.-J. (2011). Discharge Reaction Mechanism of Room-Temperature Sodium-Sulfur
Battery with Tetra Ethylene Glycol Dimethyl Ether Liquid Electrolyte. J. Power Sources..

[55] Kumar A., Ghosh A., Forsyth M., MacFarlane D. R., Mitra S. (2020). Free-Radical Catalysis and Enhancement
of the Redox Kinetics for
Room-Temperature Sodium-Sulfur Batteries. ACS
Energy Lett..

[56] Yu X., Manthiram A. (2014). Room-Temperature
Sodium-Sulfur Batteries with Liquid-Phase
Sodium Polysulfide Catholytes and Binder-Free Multiwall Carbon Nanotube
Fabric Electrodes. J. Phys. Chem. C.

[57] Kim I., Park J.-Y., Kim C., Park J.-W., Ahn J.-P., Ahn J.-H., Kim K.-W., Ahn H.-J. (2016). Sodium Polysulfides
During Charge/Discharge of the Room-Temperature Na/S Battery using
TEGDME Electrolyte. J. Electrochem. Soc..

[58] Carter R., NewRingeisen A., Reed D., Atkinson R. W., Mukherjee P. P., Love C. T. (2021). Optical Microscopy Reveals the Ambient
Sodium-Sulfur Discharge Mechanism. ACS Sustain.
Chem. Eng..

