# Supplementary material for: Metallic 1T Na x MoS2 as Sulfur Host for Room Temperature Na–S Batteries
Source: ACS Nano. 2026 May 20;20(21):15632–40. doi: 10.1021/acsnano.6c04780 (PMC13235646; doi:10.1021/acsnano.6c04780)
Supplement: Supplementary file 1 [file nn6c04780_si_001.pdf]

Supporting Information

Metallic 1T Na_xMoS_2 as Sulfur Host for Room Temperature Na-S Batteries

Esther Lilian Gray[1], Jung-In Lee[1], Alice Elizabeth Beardmore[2,3], Leyi Loh[1], Ziwei Jeffrey Yang[1], Yan Wang[1] and Manish Chhowalla[1]*

[1]Department of Materials Science and Metallurgy, University of Cambridge, Cambridge, CB30FS, UK

[2]Yusuf Hamied Department of Chemistry, University of Cambridge, Cambridge, CB21EW, UK

[3]The Faraday Institution, Quad One, Becquerel Avenue, Harwell Campus, Didcot, OX11 0RA, UK

*Corresponding author.

Email: mc209@cam.ac.uk (Manish Chhowalla)

The supplementary includes additional characterisation of the material and cathode of Na_xMoS_2. First, additional XRD spectra are shown, together with the expanded spectra of ^{23}Na magic angle spinning nuclear magnetic resonance (^{23}Na MAS NMR) and TGA of $NaBH_4$. XPS, and EDX results are provided as well as additional TEM images. Following this, the methodology used to calculate the value of x within Na_xMoS_2 is presented. Finally, the Na_xMoS_2 cathode morphology is examined by SEM and further characterised via XRD and Raman spectroscopy before and after cycling.

Additional Characterisation

Figure S1a shows the XRD pattern of Na_xMoS_2 prepared without pre-exfoliating the 2H MoS_2, showing no sodium intercalation, as indicated by the absence of the 2θ= 12.4°. Figure S1b shows the repeated batches of making Na_xMoS_2.

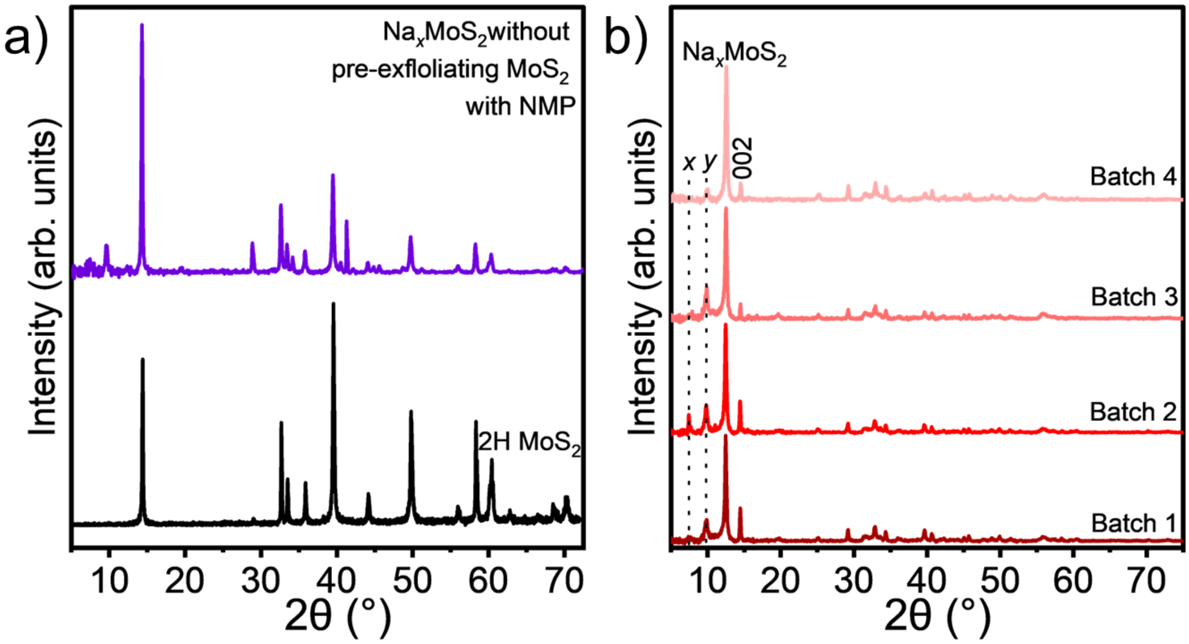

Figure S1. XRD patterns of a) Na_xMoS_2 without pre-exfoliating the MoS_2 with NMP b) Repeated batches of chemically synthesised 1T Na_xMoS_2, demonstrating reproducibility. Peaks labelled x and y indicate intercalated hydrated sodium species.

Sodium solid-state magic angle spinning nuclear magnetic resonance spectroscopy (^{23}Na MAS NMR)

Figure S2 presents the ^{23}Na magic-angle-spinning solid-state nuclear magnetic resonance (^{23}Na MAR NMR) spectra of $NaBH_4$ (blue) and synthesised Na_xMoS_2 (red). The full spectral range in Figure S2a confirms that no additional resonances are present beyond those highlighted in the zoomed-in view in the main text (Figure 3). An expanded view between +250 to –250 ppm reveals distinct spinning sidebands, marked with asterisks(*). These sidebands are characteristic artefacts of MAS NMR and are assigned based on their symmetrical spacing relative to the central peaks, consistent with the 40 kHz spinning speed used during acquisition. Figure S2c shows the expanded view from +75 to –75 ppm, showing the data plotted on a linear scale.

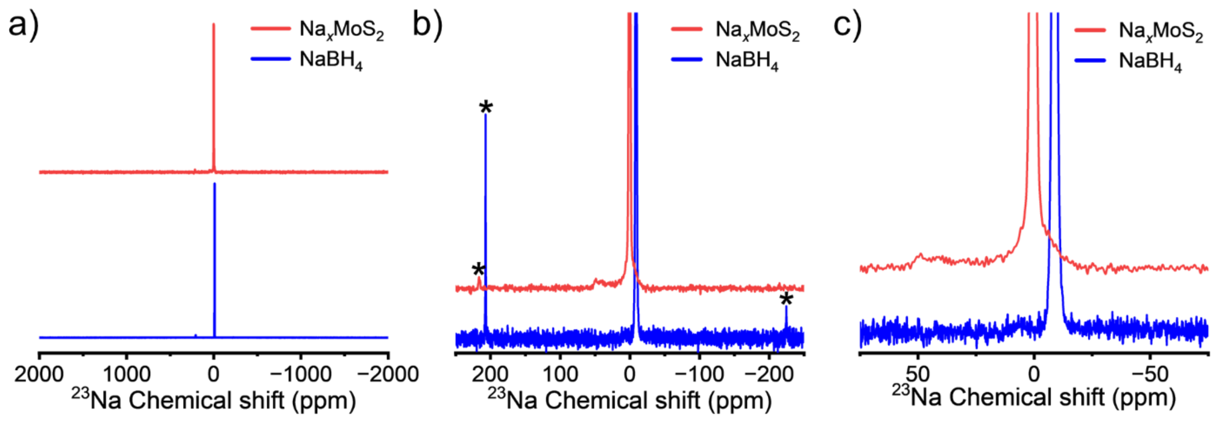

Figure S2. ^{23}Na MAS NMR spectra of $NaBH_4$ (blue) and synthesised Na_xMoS_2 (red). a) Full spectra showing no additional peaks outside the regions of interest. b) Expanded view from +250 to –250 ppm highlighting spinning sidebands (indicated by *), which are typical of MAS NMR. Sidebands are consistent with the 40 kHz spinning speed used during acquisition. c) Expanded view from +75 to –75 ppm showing the data plotted on a linear intensity scale.

Thermogravimetric analysis (TGA)

Figure S3 presents thermogravimetric analysis (TGA) of the $NaBH_4$ used in the chemical synthesis of Na_xMoS_2, performed to quantify the water content or extent of hydration within the sample. A mass loss corresponding to 8.0% was observed, indicating the presence of hydrated $NaBH_4$ when reagent is exposed to atmosphere. [1]

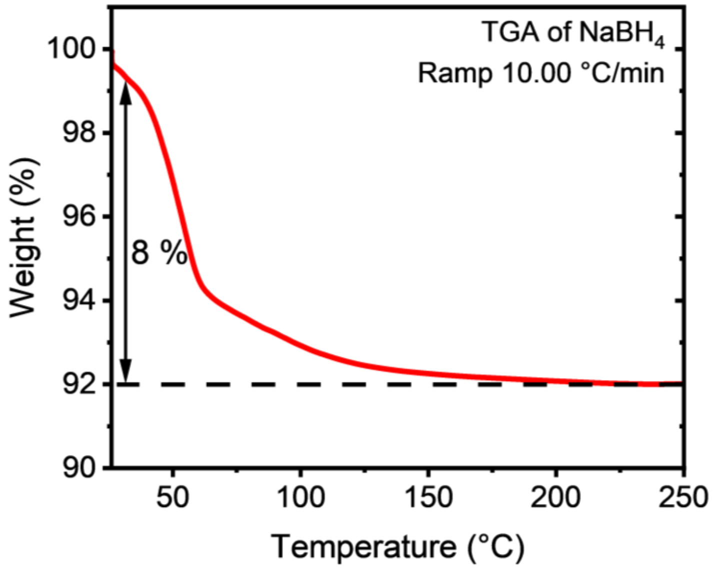

Figure S3. TGA of $NaBH_4$ used in the synthesis of Na_xMoS_2, showing a mass loss corresponding to 8.0% water content, indicating the presence of hydrated $NaBH_4$.

Boron signal X-ray Photoelectron Spectroscopy (XPS)

Figure S4 presents XPS spectra of the Na 1s, and B 1s regions. The Na 1s signal is dominated by Na^+ ions, while a minor peak from residual with $NaBH_4$. This indicates that most of $NaBH_4$ precursor was effectively removed during the three-step washing process.

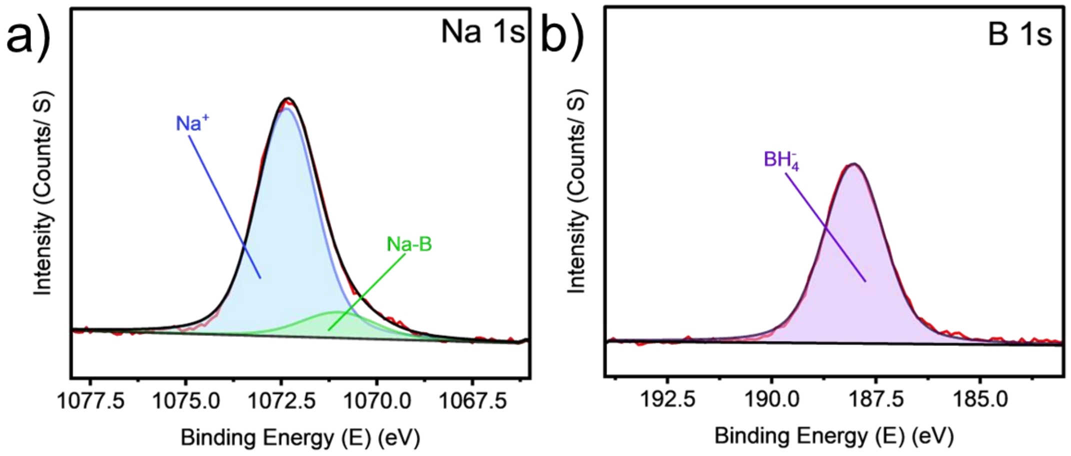

Figure S4. a) Na 1s, dominated by Na^+ ions with a minor Na-B contribution and b) B 1s, displaying a surface BH_4^- signal, indicating residual $NaBH_4$ on the material.

Boron signal Energy Dispersive X-ray Spectroscopy (EDX)

Figure S5 provides EDX of Boron peaks at three different SEM areas. All three spots indicate no or minimal is Boron present.

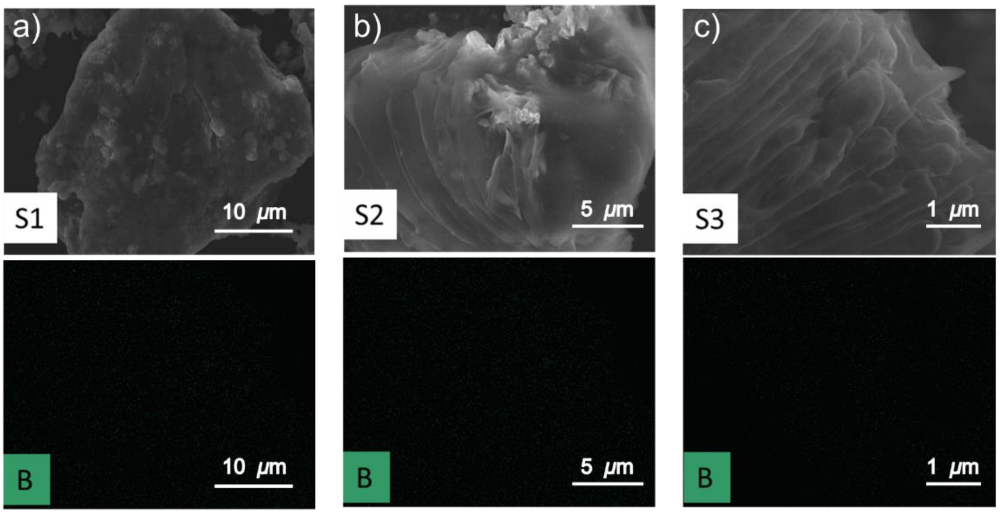

Figure S5. EDX spectra from three different SEM areas, showing no detectable or minimal boron peaks in any of the measured spots.

High-resolution Transmission Electron Microscopy (HR TEM)

Figure S6a-d present HR TEM images from multiple regions across the sample, showing the different phases (2H, yellow and 1T, grey) across each region. The area of each phase within the region was approximately quantified to estimate the relative proportions of the 2H and 1T phases, demonstrating consistent phase transformation throughout the material.

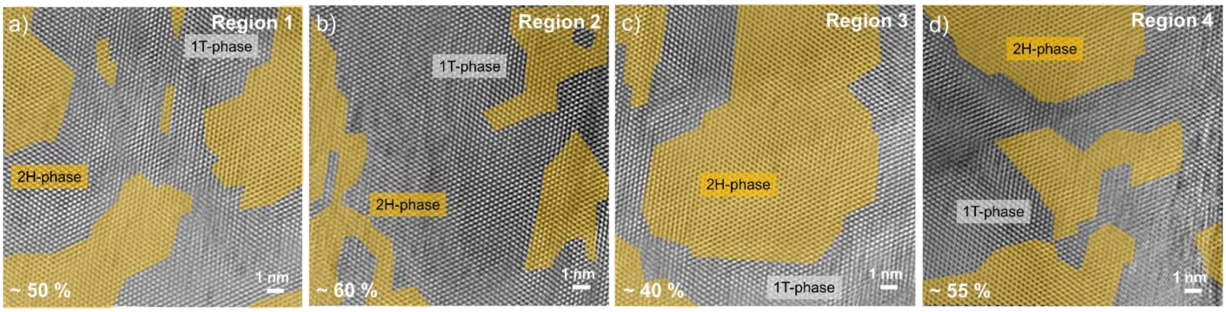

Figure S6. a-d) Additional HR TEM images of chemically sodiated Na_xMoS_2 collected from four randomly selected regions, complementing Figure 4a. Quantitative analysis of these areas confirmed that approximately 40-60% of the material consists of the metallic 1T phase, demonstrating consistent phase transformation across multiple regions.

1T Phase X-ray Photoelectron Spectroscopy (XPS)

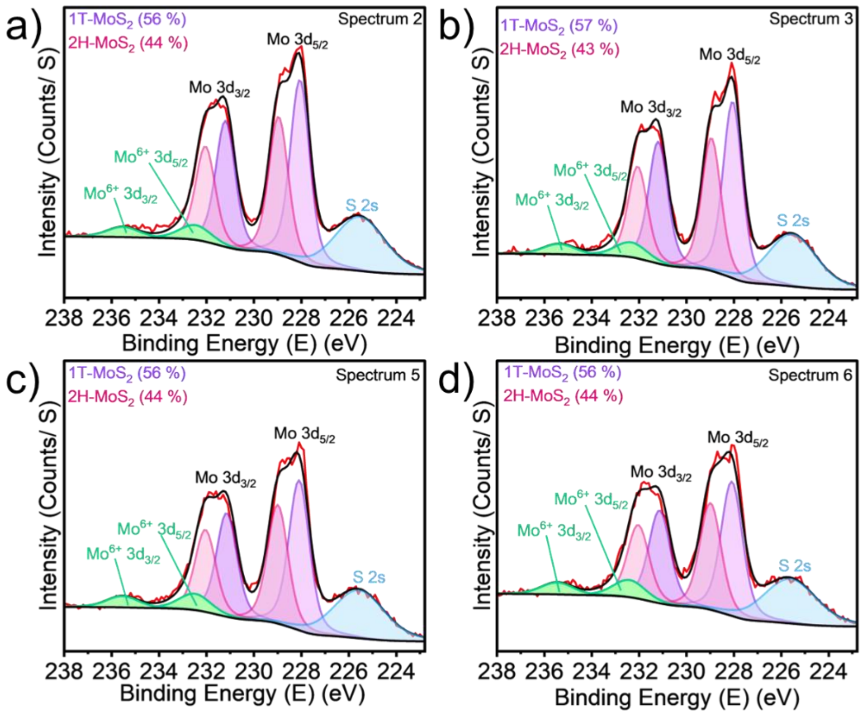

Figure S7. Mo 3d XPS spectra (point 2, 3, 5 and 6 from Figure 6a) collected from the central region of chemically sodiated 1T MoS_2. a-d) Fitted Mo 3d spectra from individual points along the central column. Across all spectra, the 1T phase constitutes approximately 56–57% of the signal.

Figure S8 presents XPS spectra of the S 2p region, presenting the coexistence of both 1T and 2H phases**.**

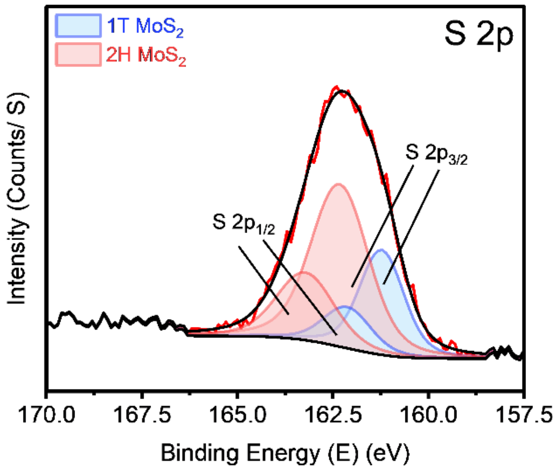

Figure S8. XPS spectra of a) S 2p, showing the presence of both 1T and 2H.

Scanning electron microscopy (SEM) and Energy Dispersive X-ray Spectroscopy (EDX)

SEM magnification images (Figures S9a–d) reveal the presence of interlayer voids and partially delaminated sheets, which further support the structural disruption expected from chemical sodiation.

Notably, in Figure S9c compared to Figure S9b and d, the edges of the layers appear more rounded, suggesting oxidation around the stacked regions. In contrast, the sharper-edged layers. This observation is indicated via Energy-Dispersive X-ray (EDX) analysis (Figure S10) of the same area, which shows higher oxygen content in the areas with rounded layers.

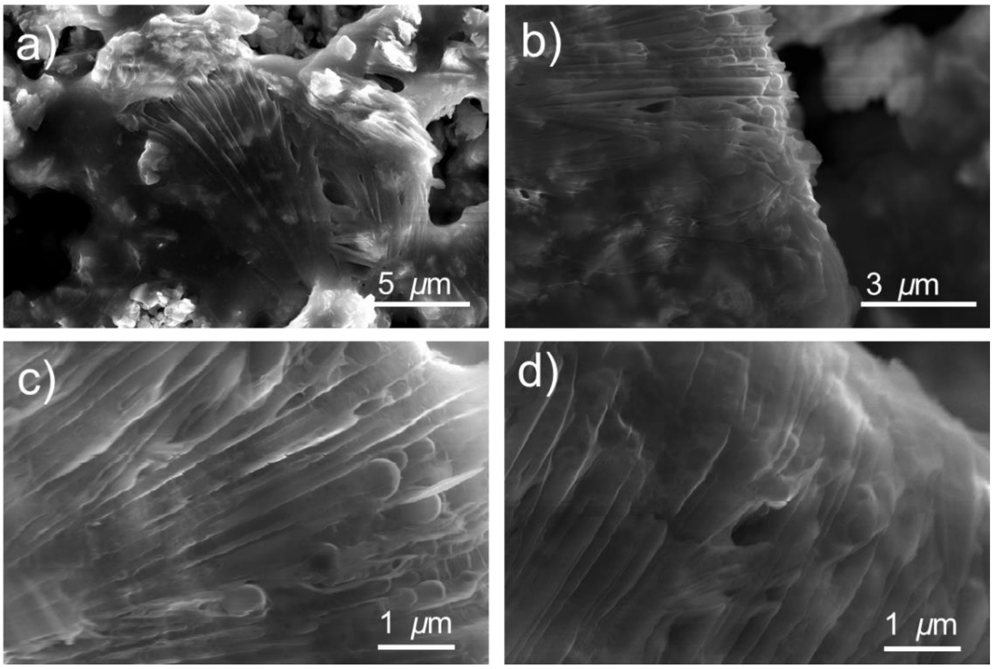

Figure S9. Scanning Electron Microscopy (SEM) of chemically sodiated 1T MoS_2. a, b) Low-magnification SEM images showing a stacked, layered morphology. c, d) Higher-magnification images highlighting interlayer voids and partial delamination of sheets. Rounded layer edges in (c) suggest localised oxidation compared to the sharper features in (d).

Energy-dispersive X-ray spectroscopy (EDX) analysis was performed on the DMF-washed Na_xMoS_2 to characterise the elemental distribution and morphology. Figure S10a show top-view SEM image of the layered material, revealing the stacked architecture typical of intercalated MoS_2, while Figure S10b presents a side-view, confirming the layered structure.

The corresponding EDX elemental maps demonstrate uniform distributions of molybdenum (Mo) and sulfur (S), indicating preservation of the layered MoS_2 framework. Sodium (Na) is consistently detected across all regions, supporting successful intercalation. The oxygen (O) is present throughout due to exposure to the atmosphere. Notably, the signal in Figure S9b appears more intense at the rounded edges of the material, which may indicate partial oxidation of sodium at these sites. This local oxidation could explain the transition from the characteristic sharp-edged morphology to the more rounded layer features observed in the SEM images. In addition, the lack of Boron (B) signal confirms sufficient washing of $NaBH_4$ with the material.

This analysis confirms that the chemically sodiated Na_xMoS_2 retains its layered morphology and uniform elemental composition, while highlighting potential surface oxidation effects arising from air exposure.

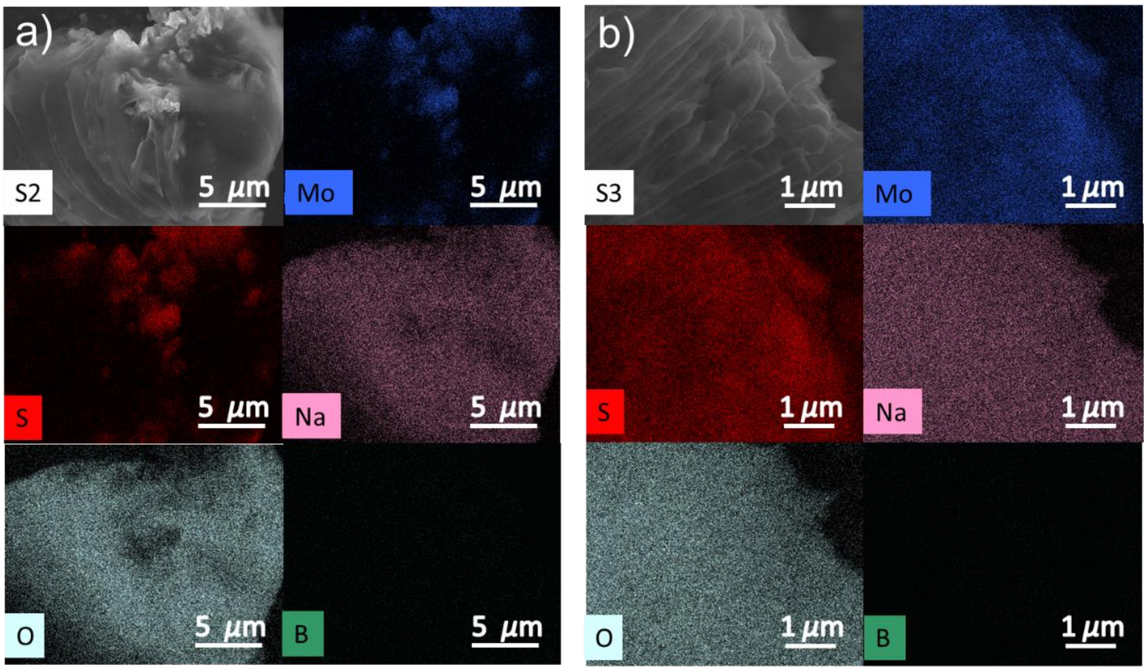

Figure S10. SEM images and EDX element mappings. a) Top-view SEM image highlighting the stacked layered morphology; b) side-view SEM image showing the cross-sectional stacking. All SEM images have corresponding EDX elemental maps for Mo (blue), S (red), Na (pink), O (light blue) and B (green) for each SEM image. Mo and S signals confirm uniform layered distribution, while Na is consistently detected throughout the sample. The oxygen signal, attributed to atmospheric exposure, appears especially intense at the rounded edges in b), suggesting partial oxidation of sodium at those sites.

EDX mapping of the unwashed Na_xMoS_2 sample (Figure S11) shows the TEM image alongside elemental distributions of Mo, S, Na, B, and O (Figure S11a–f). A distinct B signal (Figure S11e) confirms the presence of residual boron-containing species originating from the synthesis process. In contrast, the absence of boron in the DMF-washed Na_xMoS_2 (Figure S5 and S10) validates the washing procedure's effectiveness in removing $NaBH_4$. This comparison supports the conclusion that no boron in washed samples is genuine and not detection limits of the EDX.

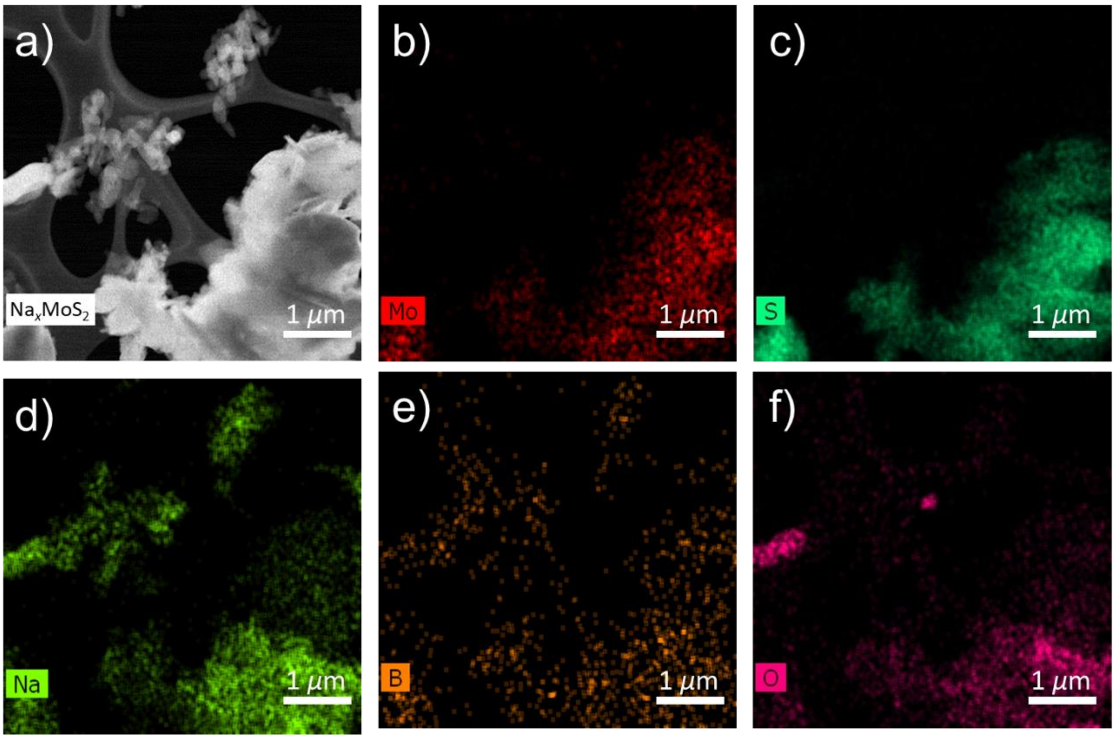

Figure S11. TEM image and EDX elemental mapping of an unwashed Na_xMoS_2 sample. a) SEM image of the material; b–f) corresponding EDX maps showing distributions of Mo, S, Na, B, and O, respectively. A clear boron signal (e) confirms the presence of residual boron-containing species from the synthesis process, highlighting the effectiveness of DMF washing in removing these species in washed samples.

Quantifying sodium in Na_xMoS_2

Deintercalation of Na_xMoS_2 was investigated to estimate the sodium content, x, in the material, as illustrated schematically in Figure S12a. The cathode was fabricated using a 70:15:15 mass ratio of Na_xMoS_2, carbon black (super-P), and polyvinylidene fluoride (PVDF) in N-methyl-2-pyrrolidone (NMP). To facilitate deintercalation, a constant current of 10 μA was applied (Figure S12b), driving the extraction of sodium ions and allowing plating onto a gold-plated stainless steel substrate. Voltage was monitored throughout the process (Figure S12c), with the experiment terminated at 2.60 V, beyond which a change in the voltage profile suggested the onset of material degradation rather than continued sodium removal.

To validate this interpretation, X-ray diffraction (XRD) was performed before and after deintercalation (Figure S12d). The pristine electrode exhibited a peak at 12.8°, corresponding

to Na-intercalated MoS_2 as previously reported. [2-4] A peak at 7.5° indicates hydrated sodium (when the material is exposed to the atmosphere) in between the MoS_2 layers, assigned from ^{23}Na MAS NMR, [4] the 14.4° (002) plane of MoS_2, and the 15° indicates the Bragg reflection of 7.5° peak. After deintercalation, only the 14.4° peak remained, confirming the removal of interlayer sodium.

Quantification of x in Na_xMoS_2 was performed by integrating the charge passed during deintercalation and applying Faraday's law (Equation S1-3). The use of a Li-ion electrolyte eliminated any contribution from sodium in the electrolyte itself, ensuring accurate determination of sodium extracted solely from the electrode material.

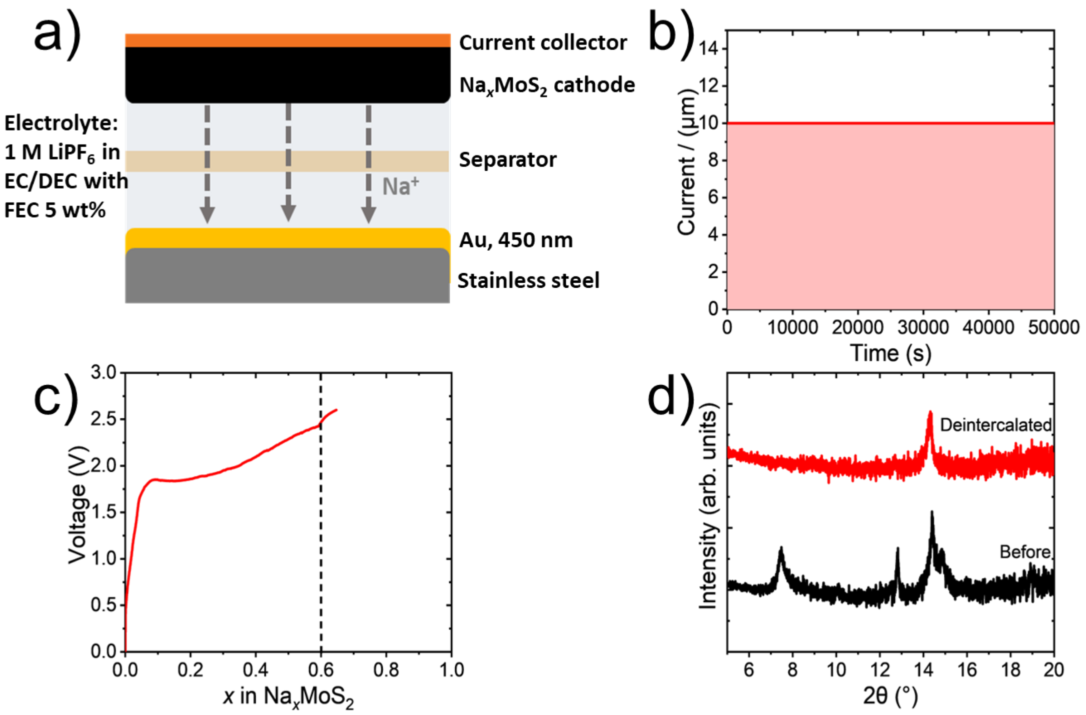

Figure S12. a) Schematic of the electrochemical setup for sodium deintercalation from Na_xMoS_2.b) Constant current (10 μA) deintercalation and corresponding. c) Voltage profile, terminated at 2.6 V to avoid material degradation. d) XRD patterns before and after deintercalation showing loss of sodium-related peaks and retention of the MoS_2 (002) peak at 14.4°, confirming sodium removal. From this data sodium content (x) was calculated using Faraday's law.

To determine the sodium content (x) in Na_xMoS_2, the total charge passed during electrochemical deintercalation was related to the known electrode mass using Faraday's law (Equation S1-3). A constant current of 10 μA was applied for 50,000 seconds, yielding a total charge of 0.5 C. This corresponds to 5.18×10^{-6} mol of deintercalated Na^+. The electrode mass was measured as 1.50 mg, and the molar mass of Na_xMoS_2 was expressed as a function of x:

$$M\,(x) = 22.99x + 160.09$$

Equation S1

Using this expression, the number of moles of Na_xMoS_2 was calculated as:

$$\frac{0.0015}{M\,(x)}$$

Equation S2

Substituting into Faraday's law, and solving for x using:

$$x = \frac{mol\ Na}{mol\ Na_xMoS_2} = \frac{5.18\ x\ 10^{-6}}{0.00150/(22.99x + 160.09)}$$

Equation S3

resulted in a sodium stoichiometry of $x \approx 0.60$. This confirms the deintercalated sample composition as approximately $Na_{0.6}MoS_2$ and demonstrates the quantitative estimation of sodium content from electrochemical measurements.

Electrochemical Performance

The S/Na_xMoS_2 cathode morphology used in Na-S batteries were analysed via SEM shown in Figure S13. The morphology appears consistent across the electrode surface, indicating uniform cathode fabrication.

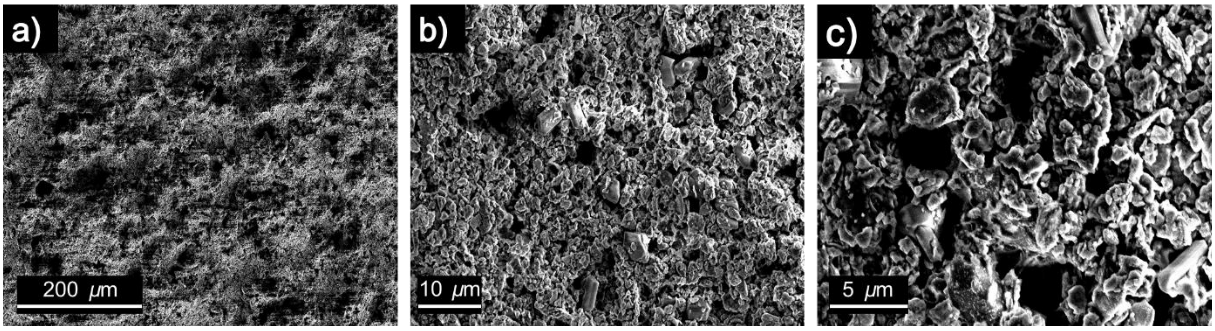

Figure S13. SEM images of the S/Na_xMoS_2 cathode at different magnifications using a scale bar of a) 200 μm, b) 10 μm, and c) 5 μm.

X-ray diffraction (XRD) and Raman data of 1T Na_xMoS_2 cathode material used before and after cycling in Na-S batteries are displayed in Figure S14a, b and c, showing characteristic peaks of the material as reported within the manuscript, confirming 1T MoS_2 stability within the Na-S battery after 10 cycles.

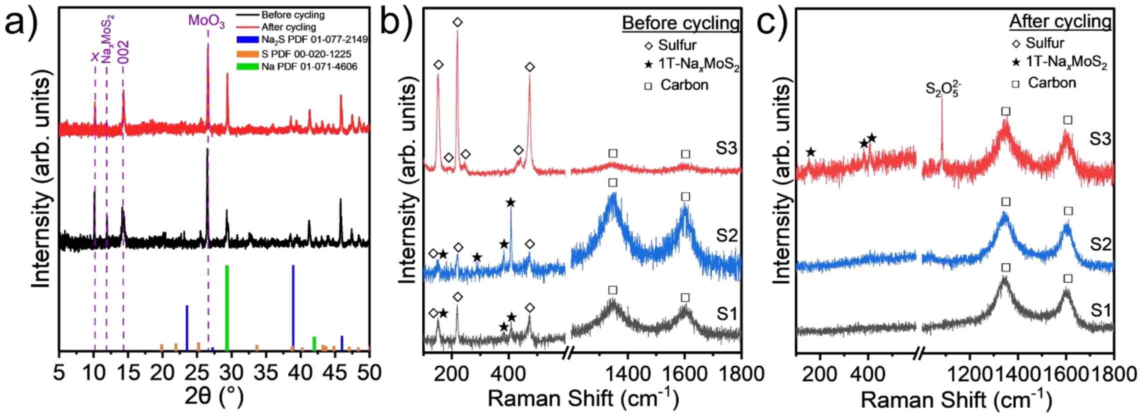

Figure S14. a) X-ray diffraction (XRD) b) and c) Raman spectra of the 1T Na_xMoS_2 cathode before and after 10 cycles. The XRD patterns show no emergence of new interlayer spacing and retain the characteristic peaks of 1T Na_xMoS_2 after cycling, indicating preservation of the host structure. The Raman spectra retain the characteristic J_1 peak, along with the E_{2g}^1 and A_{1g} modes after cycling, suggesting that the 1T phase remains intact and that no significant structural degradation has occurred.

A comparative electrochemical evaluation of the Na_xMoS_2 cathode in the Li-S and Na-S configuration is shown in Figure S15. The Li-S system delivers stable electrochemical behaviour, despite the presence of intercalated sodium not being intrinsically advantageous for this chemistry.

In contrast, the Na-S system exhibits a pronounced decrease in coulombic efficiency from 93 % to 59 % between cycles 1 and 2. Most capacity loss occurs within the initial cycles. This behaviour reflects irreversible reactions and the sensitivity of RT Na-S electrochemistry to interfacial and electrolyte conditions. Following this stage, the coulombic efficiency stabilizes, suggesting predominantly reversible redox behavior in subsequent cycles.

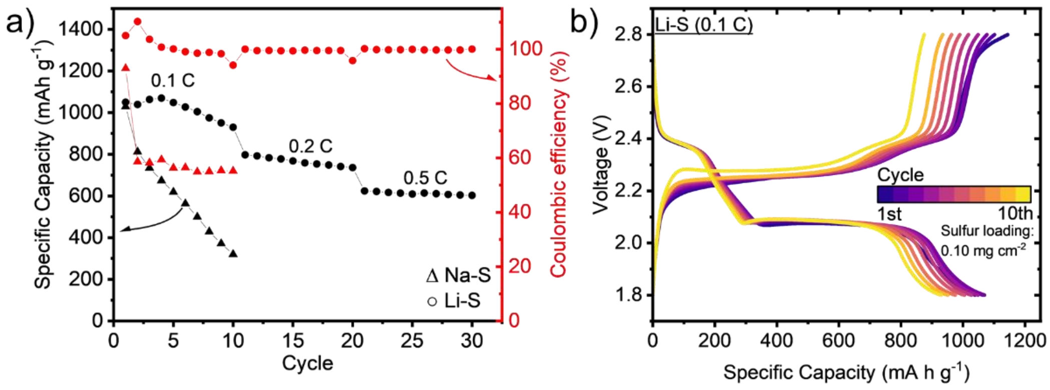

Figure S15. Cycling performance of the Na_xMoS_2/S cathode. a) The coulombic efficiency and specific capacity of Na-S and Li-S systems b) GDC of the same cathode in a Li-S battery, showing stable e specific capacity and cycling stability (1079 to 929 mAh g^{-1} over 10 cycles at 0.1 C), highlighting the catalytic potential of the Na_xMoS_2 material as a sulfur host in a well-established system.

References

(1) Luo, X.; Rawal, A.; Aguey-Zinsou, K.-F. Evidence of Superionic Na^+ Conductivity in Partially Hydrolyzed $NaBH_4$. *J. Phys. Chem. C.* **2024**, *128*, 14861-14870. DOI: 10.1021/acs.jpcc.4c01191.

(2) Wang, X.; Shen, X.; Wang, Z.; Yu, R.; Chen, L. Atomic-Scale Clarification of Structural Transition of MoS_2 upon Sodium Intercalation. *ACS Nano* **2014**, *8*, 11394-11400. DOI: 10.1021/nn505501v.

(3) Cook, J. B.; Ko, J. S.; Lin, T. C.; Robertson, D. D.; Kim, H.-S.; Yan, Y.; Yao, Y.; Dunn, B. S.; Tolbert, S. H. Ultrafast Sodium Intercalation Pseudocapacitance in MoS_2 Facilitated by Phase Transition Suppression. *ACS Appl. Energy Mater.* **2023**, *6*, 99-108. DOI: 10.1021/acsaem.2c02368.

(4) Zheng, J.; Zhang, H.; Dong, S.; Liu, Y.; Tai Nai, C.; Suk Shin, H.; Young Jeong, H.; Liu, B.; Ping Loh, K. High Yield Exfoliation of Two-Dimensional Chalcogenides using Sodium Naphthalenide. *Nat. Commun.* **2014**, *5*, 2995. DOI: 10.1038/ncomms3995.
